# FlexESI: An Automated Vapor-Switching Interface for Enhanced Flexibility and Sensitivity in Electrospray Ionization

**DOI:** 10.1021/acsmeasuresciau.5c00086

**Published:** 2025-10-08

**Authors:** Ying-Rong Hwang, Decibel P. Elpa, Pawel L. Urban

**Affiliations:** Department of Chemistry, 34881 National Tsing Hua University, 101, Section 2, Kuang-Fu Rd., Hsinchu 300044, Taiwan

**Keywords:** electrospray ionization, interface, ion source, mass spectrometry, sensitivity, signal enhancement, vapor

## Abstract

One of the ways to improve the performance of electrospray ionization (ESI) mass spectrometry (MS) is to introduce additives to the sample solution. Alternatively, such additives can be introduced in the gaseous form directly into the electrospray plume. Normally, only one additive can be introduced via one method. Here, we present a flexible automated system that enables dynamic switching among several gas-phase additives, which can alternately be introduced to the ion source compartment over a short period of time. These additives include vapors of acids and solvents. We show that different gaseous additives enhance the signals of different analytes to a varied extent. The enhancement factors were in the range ∼ 2–15. We applied the gas-phase additive switching in real sample analysis, where it enhanced signal intensities and broadened the detection range. The automated system also enables the dosing of vapors at different concentrations. Unlike conventional approaches that saturate the electrospray plume with a fixed vapor level, controlled dosing of acid or solvent vapor levels enables optimization of the protein signal intensity and facilitates structural probing. Overall, it is possible to systematically vary both the type and the concentration of vapor additives using a single setup, improving the analytical performance and versatility of ESI-MS. The proposed setup is compatible with liquid chromatography.

## Graphical abstract

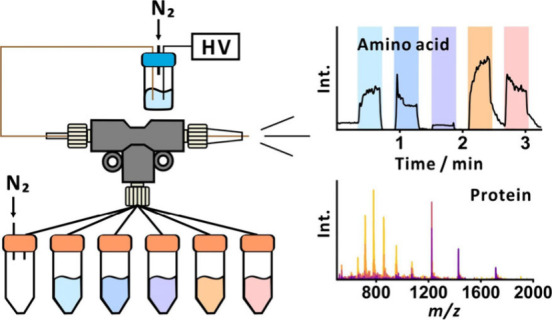

## Introduction

Electrospray ionization (ESI) is a robust and sensitive atmospheric pressure ionization technique that produces gas-phase ions with minimal fragmentation.^1^ Through ESI, intact biologically important large molecules such as proteins are ionized by multiple charging.^2^ Hence, ESI-mass spectrometry (MS) is the analytical technique of choice for analyzing a broad range of polar, nonvolatile, and thermally labile compounds in complex matrices.^1,3,4^ Although ESI-MS offers many advantages, enhancing analyte detection and sensitivity remains a key focus of research. Various factors affect ionization efficiency such as analyte physicochemical properties (*e*.*g*. p*K*a, polarity, molecular weight), solvent, sample matrix, and flow rate.^1,5–7^ To produce gas-phase ions effectively from the analyte molecules in solution, chemical additives are generally used. For instance, the analyte response is enhanced by simply adding an acid or a base to the sample solution or mobile phase to aid in the protonation or deprotonation of analytes in the solution.^1,8^ Moreover, for analytes in a mixture, solvent systems—with different strengths of organic solvents, organic modifiers, and buffer concentrations—provide varying degrees of ionization enhancement.^9^

In recent years, researchers have explored altering the ESI spray environment using various chemical vapors.^10–12^ These vapors can be easily introduced into the ESI source by redirecting the instrument’s nebulizer or drying gas through additional containers filled with acids, bases, or alcohols.^13,14^ Modification of nitrogen gas to expose electrospray droplets to chemical vapors has been primarily used in protein and peptide research. For example, manipulating the charge state distribution (CSD) of proteins can be achieved by adjusting the acidity or basicity of the vapor environment.^15,16^ Additionally, chemical vapors are used to enhance the analyte signals. Some research teams have demonstrated that vapors of acids or organic solvents effectively enhance analyte signals and reduce background interference in detecting low-abundance proteins and peptides.^11,14,17,18^ The use of vapors to modify the ESI environment has gained increasing interest because—unlike traditional postcolumn solvent addition—this approach offers several advantages, including minimization of solvent consumption, reducing sensitivity loss due to dilution, and suitability for coupling with a separation technique that requires long analysis time such as liquid chromatography.^10,14^ Moreover, the operation of ESI at nanoliter flow rates (<1000 nL min^–1^) has been a pivotal development over conventional ESI.^19–22^ Nanoelectrospray ionization (nanoESI) produces smaller droplets than conventional ESI, leading to faster desolvation.^21^ NanoESI also offers enhanced tolerance to salt contamination and minimizes analyte suppression in complex mixtures.^23^

Smart experimental systems that handle noncognitive and repetitive tasks are becoming increasingly popular in chemistry research.^24^ A wide range of affordable electronic modules, such as microcontroller boards and single-board computers, are easily accessible even to the general public.^25^ With programmed control, these electronic components can operate automatically, achieving full automation of the analytical workflow. For instance, the microcontroller manages various functions, including motor control, instrument triggering via relays, and solenoid valve actuation.^26^ Those systems can speed up the research process, enhance reproducibility, and reduce human effort.^27–29^

Exposing the electrospray or nanoelectrospray plume to different vapors has been reported, using one vapor additive in each analysis.^10,14,18,30,31^ Here, we demonstrate FlexESI – an automated system for dynamic switching of gas-phase additives in nanoESI-MS to boost analytical flexibility and sensitivity. Introducing multiple vapor additives in a single analytical run is a promising strategy, particularly for detecting analytes in biological fluids or sample extracts, where sample volumes are often limited. These gas-phase additives are alternately introduced into the ion source compartment by a system of tubings fitted with valves. The six-channel switchable vapor delivery system enables sequential and gradient analyses with different additives.

## Experimental Section

### Chemicals

Cytochrome *c* (90%, from horse heart muscle), formic acid (>98%), l­(+)-aspartic acid (98+%), l­(+)-glutamic acid (99%), l-histidine (98%), l­(−)-tryptophan (99%), and l-valine (99%) were purchased from Acros Organics (Geel, Belgium). Choline chloride (98+%), D-fructose (99%), l-proline (99%), l-tyrosine (99%), and propionic acid (99%) were purchased from Alfa Aesar (Ward Hill, MA, USA). Five custom-synthesized peptides, including (G: glycine; A: alanine; Y: tyrosine; L: leucine; F: phenylalanine) GGG, GGA, GGY, GGL and GGF (≥95%), were purchased from ABclonal (BioAB; New Taipei, Taiwan). Acetone (LC-MS grade), acetonitrile (LC-MS grade), and water (LC-MS grade) were purchased from Fisher Scientific (Waltham, MA, USA). d-(+)-glucose (≥99.5%), l-lysine (≥98.0%, TLC), and l-phenylalanine (98.5%–101.0%) were purchased from Sigma-Aldrich (St. Louis, MO, USA). Methanol (LC grade) and 2-propanol (LC-MS grade) were purchased from Merck (Darmstadt, Germany). Acetic acid (≥99.8%) was purchased from Honeywell (Charlotte, NC, USA). Ethanol (>99.5%, anhydrous), was purchased from Echo Chemical (Miaoli, Taiwan). Ubiquitin (recombinant human, >95%) was purchased from R&D Systems (Minneapolis, MN, USA). Longan flower honey was purchased from a local retail store (Hsinchu, Taiwan). For the analyses using sequential vapor introduction, the individual sample solutions for amino acids and peptides were 5 μM mixture of six amino acids (aspartic acid, lysine, glutamic acid, histidine, tyrosine, tryptophan) in 25% (v/v) aqueous methanol solution with 0.1% (v/v) acetic acid and 5 μM mixture of five peptides (GGG, GGA, GGY, GGL, GGF) in 25% (v/v) aqueous methanol solution with 0.1% (v/v) acetic acid, respectively. For the analyses using gradient vapor introduction, the individual sample solutions for ubiquitin and cytochrome *c* were 10 μM ubiquitin dissolved in a 10% (v/v) aqueous methanol solution with 1 mM ammonium acetate and 10 μM cytochrome *c* dissolved in a 10% (v/v) aqueous methanol solution with 1 mM ammonium acetate, respectively.

### Vapor Control Setup

The 6-channel vapor delivery system (Figure 1A) was designed to introduce different vapors into the nanoESI source at specific time points. An electronic control system was used for vapor introduction and the MS trigger (Figure S1). The electronic components of the 6-channel vapor delivery system included a function generator (Analog Discovery 2; part no. 210–321; Digilent, Pullman, WA, USA), an 8-channel relay module (part. no. 119312, Centenary Materials), a DC-DC converter (part. no. 96038; Centenary Materials), and six pinch valves (2-way NC, 1/32″ ID × 3/32″ OD, 12 V, response 25 ms, max pressure 15 psi; PreciGenome, San Jose, CA, USA). A 15 mL conical tube (material: polypropylene; LabServ, Fisher Scientific, Waltham, MA, USA) was used as a reservoir for 6 mL of each vapor additive. All chemical additives were used pure except 75% (v/v) aqueous formic acid solution.

**Figure 1:**
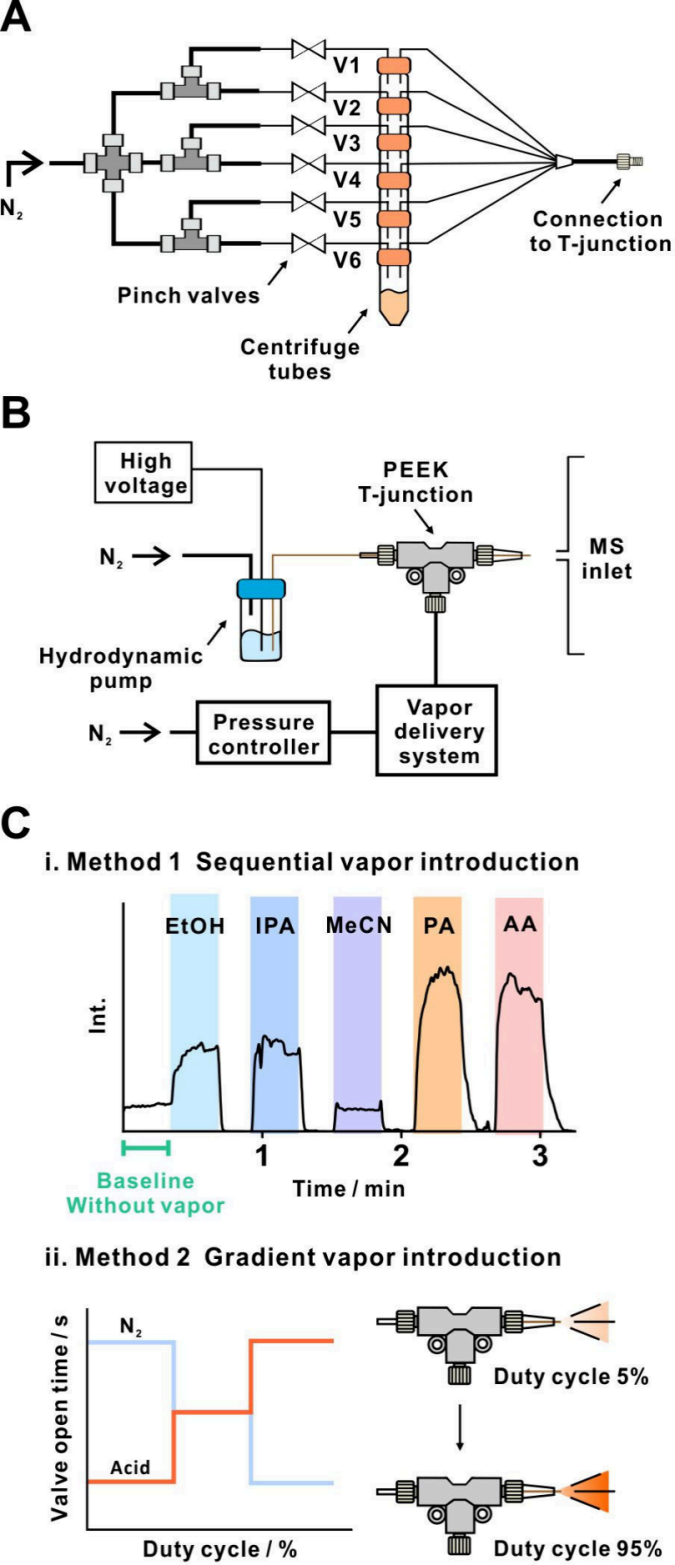
Setup for automated switching of gas-phase additives to nanoESI-MS: (A) 6-channel vapor delivery system; (B) homemade nanoESI setup; (C) two methods of vapor introduction: (i) sequential and (ii) gradient.

The controlled introduction of each vapor was achieved by passing nitrogen gas, which was delivered via a 1/8 in. polytetrafluoroethylene (PTFE) tubing (length, 3 cm; ID, 1.5 mm; OD, 3.2 mm; Supelco, Bellefonte, PA, USA), through a pressure controller (IQP-600C, Bronkhorst, Ruurlo, Netherlands). The gas stream was then split into six separate flows using a 1/8-in. union cross (material: stainless steel; part no. SS-200–4; Swagelok, Solon, OH, USA) and then three 1/8-in. union tees (material: stainless steel; part no. SS-200–3; Swagelok). Each of the six gas streams flowed through a 1/8-in. PTFE tubing (length, 10 cm; Supelco). This tubing was then connected to a 1/16-in. PTFE tubing (ID, 0.8 mm; Supelco) for direct connection to the 1/16-in. silicone tubing inlet (length, 4 cm; ID, 0.8 mm; OD, 2.5 mm; Beion, Shanghai, China) of the pinch valves. Note that for this PTFE tubing, its length (3.5–8.0 cm) was adjusted for the spatial arrangement of each vapor channel. The silicone tubing outlet of the pinch valve was connected to the 1/16-in. PTFE inlet tubing (length, 19 cm; Supelco) fixed inside each 15 mL vapor reservoir. Another 1/16-in. PTFE tubing (length, 7 cm; Supelco), fixed inside the vapor reservoir, served as the vapor outlet. The tips of both the inlet and outlet PTFE tubing were positioned at a depth of 3 cm inside the conical tube reservoir. Each PTFE vapor tubing outlet was then connected to a longer 1/16 in. PTFE tubing (length, 28.5 cm; Supelco) via a section of silicone tubing (length, 1 cm; ID, 0.8 mm; OD, 2.5 mm; Beion, Shanghai, China). Finally, all six 1/16 in. PTFE tubings were merged into a single vapor outlet using a modified 200-μL pipet tip (Eppendorf, Hamburg, Germany), allowing the gas flow to enter the nanoESI source via a 1/8 in. PTFE tubing (length, 3 cm; Supelco). To ensure airtight connections between PTFE tubings of different sizes, silicone tubings with appropriate inner and outer diameters were used (Figure S2).

### NanoESI Source with Switchable Vapor Delivery System

A house-built nanoESI setup (Figure 1B) was used for this study. A fused silica capillary emitter (length, 6 cm; ID, 0.02 mm; OD, 0.38 mm; part no. 1010–31442; GL Sciences, Tokyo, Japan) was passed through the two coaxial ports of a PEEK tee (thru hole, 0.04 in part no. P-714; IDEX Health & Science, Rohnert Park, CA, USA). One port was fitted with a 1/16-in. PTFE tubing (length, 3 cm; ID, 0.3 mm; OD, 1.6 mm; Supelco) to secure the capillary emitter in place while another port was fitted with a 1/16-in. PTFE tubing (length, 2 cm; ID, 0.8 mm; OD, 1.6 mm; Supelco), allowing the chemical vapor to be delivered from this end. An outer tip (length, 15 mm; ID, 2.5 mm) was fabricated by cutting a 200-μL pipet tip and then fitted over the capillary emitter and 1/16-in. PTFE tubing to promote mixing of vapor with the nanoESI spray. The capillary emitter was protruded ∼1 mm beyond the pipet tip. The other port of the PEEK tee was connected to the vapor delivery system via PTFE tubing (length, 3 cm; ID, 1.5 mm; OD, 3.2 mm; Supelco) and a fitting. The sample solution was delivered to the capillary emitter using a hydrodynamic pump (Figure S3).

### Mass Spectrometry

All analyses of small molecule and protein sample solutions were performed by using a triple quadrupole (QqQ) mass spectrometer (LCMS-8030; Shimadzu, Kyoto, Japan). The distance between the nanoESI emitter and the MS inlet was set to 5 mm, and a high voltage of +4.0 kV was applied to the nanoESI solution. Note that a relatively high potential was applied because of a significant voltage drop within the capillary connecting electrolyte vial and emitter (*cf*. refs. 32,33 ). The heat block temperature was 250 °C and the desolvation line temperature was 250 °C. Drying gas was not used to avoid signal loss in nanoESI. The mass analyzer was operated in multiple reaction monitoring (MRM) mode for amino acid and peptide analyses under 230 kPa collision-induced dissociation (CID) gas pressure. The MRM transitions and collision voltages are given in Table S1. Protein analysis was performed in full-scan mode with a scan range of *m*/*z* 500–2000. The MS analysis time was 3.25 min for sequential vapor introduction and 1 min for gradient vapor introduction.

For real sample analysis, compound identification was performed using a full scan and MS/MS (product ion scan) on the QqQ-MS, with the same instrument parameters used for the analysis of small molecule sample solutions. For quadrupole time-of-flight-MS (Q-ToF-MS) (LCMS-9030; Shimadzu) experiments, data were acquired in full-scan mode and in MS/MS (product ion scan). An ESI voltage of +4.0 kV was applied. The nebulizing gas flow rate was set at 3 L min^–1^, and the heating gas flow rate at 8 L min^–1^. The interface temperature was 250 °C, and the desolvation line temperature was 250 °C. CID was performed at a gas pressure of 230 kPa during the MS/MS scan.

### Vapor Introduction Workflow

We tested two methods of FlexESI (sequential and gradient delivery of different vapors) to introduce vapors of chemical additives into the nanoESI spray (Figure 1C). In the first method, vapors were automatically introduced sequentially into the nanoESI plume to evaluate their effects on the signal intensities of test analytes (amino acids and peptides) within a single MS analysis (Figure 1Ci). An equilibration step was conducted at the start of each experiment day to ensure consistent vapor levels within the flow system before analysis. Each channel was sequentially opened to flush vapors at 160 mbar (corresponding to ∼510 mL min^–1^) for 1 min, for a total duration of 5 min. This equilibration step was performed only once per day. For sequential vapor delivery, a 20-s baseline without vapor (*i*.*e*., only from nanoESI spray) was first acquired. Vapors were then introduced individually for 20 s at nitrogen gas pressures of 80 mbar (corresponding to ∼276 mL min^–1^) and 160 mbar (corresponding to ∼510 mL min^–1^) for vapors of organic solvents and acids, respectively. Between vapors, a nitrogen pressure at 650 mbar was used to flush the channels for 15 s. A washing step was incorporated into the workflow by rinsing the nanoESI flow line with a diluent of the analyte solution after three consecutive analyses.

In the second method, an acid or solvent vapor was gradually introduced by controlling the valve opening time of the vapor channel (Figure 1Cii). Here, an equilibration step was performed before starting the analysis for each vapor. The vapor channel was opened to flush the vapor at 160 mbar (corresponding to ∼510 mL min^–1^) for 1 min. For gradient vapor introduction, a 15-s baseline without vapor (*i*.*e*., only from nanoESI spray) was initially obtained, followed by an acid or solvent vapor ramp. Each step of the ramp lasted 2 s. In the first step (duty cycle, 5%), the valve for the acid channel was opened for 0.1 s, while the valve for the nitrogen channel was opened for 1.9 s. In the second step (duty cycle, 10%), the valve for the acid channel was opened for 0.2 s, while the valve for the nitrogen channel was opened for 1.8 s. With each subsequent step (increments of 5% duty cycle), the opening time for the acid channel valve gradually increased, while that of the nitrogen channel valve decreased, resulting in an increasing acid (or solvent) vapor level over 20 steps (up to duty cycle of 100%) within 40 s. The gas pressures of the acid and nitrogen channels were both set at 120 mbar (corresponding to ∼428 mL min^–1^). A higher pressure of nitrogen (650 mbar) was used to flush the vapor additive channel for 6 s after each ramp. Additionally, because protein native structures are highly sensitive to acid vapor residues, the outer tip was rinsed with water and ethanol and then dried with nitrogen gas before each analysis.

## Results and Discussion

### Optimization of Vapor Introduction into the NanoESI Source

To achieve stable MS signals when evaluating the effects of vapor additives on the test analytes, we optimized the nitrogen gas pressure and sample flow rate. The amount of vapor delivered into the nanoESI spray depends on the nitrogen gas pressure supplied to the vapor delivery channels. Here, we varied nitrogen gas pressures (80–160 mbar) for five vapor additives (ethanol (EtOH), isopropanol (IPA), acetonitrile (MeCN), propanoic acid (PA), and acetic acid (AA)) to analyze a mixture of amino acids in solution (Figure S4A-C). Four of the vapor additives, except MeCN, showed increased amino acid signal intensities across all nitrogen gas pressures compared to signals without vapor (*i*.*e*., only from the nanoESI spray). However, MeCN consistently caused signal suppression for all analytes. For PA and AA, signal intensities were at a maximum when the nitrogen gas pressure was 160 mbar (Figure S4C). On the other hand, EtOH and IPA exhibited the highest signal intensity at 80 mbar (Figure S4A). Considering that organic polar solvents and short-chain carboxylic acids exhibit distinct physical and chemical properties—such as volatility, vapor pressure, and proton affinity—the optimum conditions for vapor introduction are influenced by these properties.^10,34,35^ Therefore, we selected 80 and 160 mbar as the optimum nitrogen gas pressures to introduce vapors of organic solvents and acids, respectively.

Additionally, the sample flow rate was optimized (Figure S4D–F; for the relationship between the applied pressure and flow rate, refer to Figure S5). Both low (75 nL min^–1^) and moderate (100 nL min^–1^) sample solution flow rates resulted in signal instability and in-between run deviations for most analytes across different vapor additives (Figure S4D,E). In contrast, a stable and high signal intensity was achieved when the flow rate was increased to 129 nL min^–1^ (Figure S4F). Consequently, to minimize signal fluctuations and achieve optimum signal enhancement, a sample solution flow rate of 129 nL min min^–1^ was selected for analyses using sequential vapor introduction. For details on precision evaluation, refer to Figure S6, and Tables S2 and S3, and for additional discussion, refer to the Supporting Information.

### Sequential Vapor Introduction for Small Molecule Analysis

The effect of exposing solutions of small molecules to vapor additives during nano-ESI was investigated. Here, five vapor additives were automatically introduced—one at a time—within a single MS analysis (Figure 1Ci). Exposure of small molecules (amino acids and peptides) to different vapor additives exhibited varying degrees of MS signal enhancement. We tested a 5 μM mixture of six amino acids with different functional groups and observed a generally consistent trend in signal enhancement except for lysine and histidine (Figure 2A–F). The EICs of amino acids showed that PA vapor provided the highest signal enhancement, followed by AA, EtOH, and IPA. Nonetheless, MeCN vapor significantly suppressed the signal. We also calculated the enhancement factor (*EF*) for each amino acid (Table S4) to evaluate the effects of different vapors (for data processing details, see Supporting Information). Briefly, to assess the signal enhancement from the vapor additive, *EF*s were calculated using the following equation:

1 $$EF={I/I}_{0}$$

where *I*_0_ is the average signal intensity without vapor, while *I* is the average signal intensity with vapor. Among the analytes, aspartic acid and glutamic acid exhibited the highest signal enhancements under PA vapor (Figure 2A,B), with *EF* values of ∼15 and ∼11, respectively. Overall, the highest *EF*s for most amino acids were obtained upon exposure to PA vapor (*EF*s from ∼3 to 15). However, for histidine, the *EF* from PA vapor was similar to that obtained from the solvent vapors (*EF* ≈ 3).

**Figure 2:**
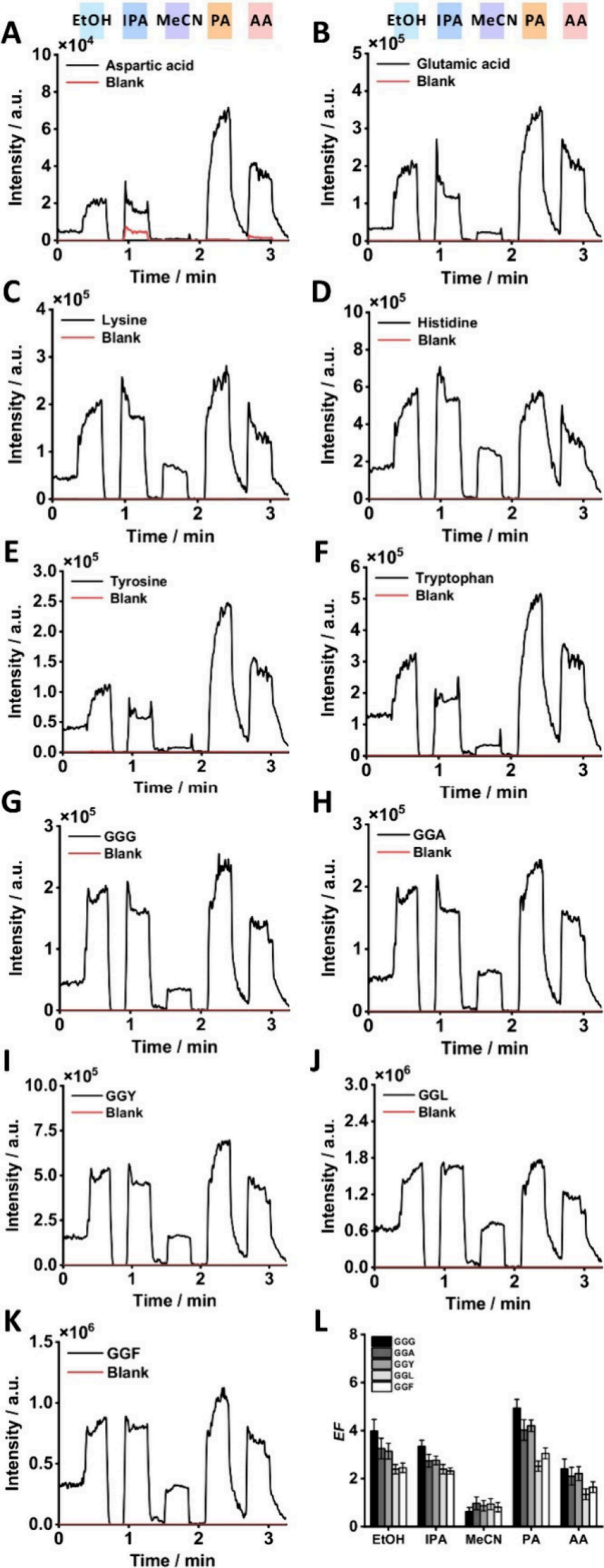
EICs of amino acids and peptides exposed to sequentially introduced vapor additives. Vapor sequence: EtOH–IPA–MeCN–PA–AA: (A) aspartic acid; (B) glutamic acid; (C) lysine; (D) histidine; (E) tyrosine; (F) tryptophan; (G) GGG; (H) GGA; (I) GGY; (J) GGL; (K) GGF; and (L) *EF*s. Sample solutions: 5 μM amino acid mixture in 25% (v/v) aqueous methanol solution with 0.1% (v/v) AA; 5 μM peptide mixture in 25% (v/v) aqueous methanol solution with 0.1% (v/v) AA. One out of three replicates is shown.

The MeCN vapor suppressed the signals of most amino acids, while lysine (*EF* = 1.24) and histidine (*EF* = 1.37) remained unaffected. Considering the possible enhancement from IPA vapor carryover (Table S5), the *EF*s are ∼1, suggesting minimal impact (for additional details on evaluation of carryover, see Figure S7, Table S5, and additional discussion in Supporting Information). This is consistent with the findings of Colizza et al., who reported that MeCN-induced ion suppression selectively affects compounds with functional groups such as carbonyls, peroxides, and triazoles, while amine-containing compounds such as hexamine were largely unaffected.^36^ Lysine and histidine—both basic, amine-containing amino acids, were less affected by MeCN vapor (Figure 2C,D).

We also analyzed a 5 μM peptide mixture under sequential vapor introduction (Figure 2G–K). Based on the EICs and *EF*s of the tested peptides, PA vapor provided the highest signal enhancement (*EF*s from ∼ 3 to 5), followed by EtOH, IPA, and AA (Figure 2G–L and Table S6). On the other hand, MeCN vapor either suppressed the peptide signal or had no effect. Given that the tripeptides share similar physicochemical properties with amino acids, the results observed with PA and MeCN were expected. The peptides used here have partitioning coefficients (*K*) in the following order: GGG < GGA < GGY < GGL < GGF, where *K* is defined as the ratio of the analyte concentration on the droplet surface to that of the droplet interior.^37,38^ In one study, the authors attributed dependency of ESI responses to the nonpolar character of peptides in an equimolar mixture.^38^ The enhanced affinity for the droplet surface allows effective competition for excess charge, enabling higher ESI response.^38^

A general trend was observed in which the *EF* values decreased with increasing nonpolar side chains of peptides (Figure 2L, Table S6). GGG, the peptide with the smallest nonpolar side chain (lowest *K* or surface affinity), showed the highest *EF*s across all vapors, except in MeCN, where its signal was suppressed. However, GGL and GGF—the two peptides with largest nonpolar side chains (highest *K*s or surface affinities)—exhibited lower *EFs* compared to GGG across all vapors, except in MeCN, where their signals were suppressed. Notably, the intensities of GGL and GGF, being more hydrophobic, were higher than those of the other peptides (GGY, GGA, GGG) in the 5 μM mixture (Figure 2J,K). Further, a previous study reported that hydrophilic compounds tend to reside in larger primary droplets which are concentrated at the center of the electrospray plume.^39^ In contrast, hydrophobic compounds are found in smaller satellite and progeny droplets, which are pushed toward the electrospray plume periphery due to their high charge density.^39^ Since *EF* was calculated as the fold change of peptide signal with and without vapor exposure, GGL and GGF, the peptides with largest nonpolar side chains (or highest surface affinities) among the test peptides, showed lower *EF*s despite effective enhancement mechanism. This is because their inherently stronger ionization relative to the other peptides resulted in higher signal intensities, even without vapor exposure.

In this study, MeCN either suppressed or had no effect on the signals of small molecules tested (Tables S4 and S6). The gas-phase proton affinity of MeCN (∼779.2 kJ mol^–1^) is relatively low compared to the gas-phase proton affinities of the small molecules tested (amino acids, ∼908.9–996 kJ mol^–1^).^40^ It was suggested that reagents with proton affinities higher than those of background ions, but lower than those of analyte ions, are ideal for suppressing background signals without affecting peptide ion signals.^12^ Based on proton affinity alone, one might expect MeCN vapor to enhance ionization by promoting proton transfer to analytes with a higher proton affinity. However, in our work, the suppression of small molecules by MeCN vapor suggests that other factors affect the enhancement mechanism. The suppression effect of MeCN in atmospheric pressure ionization MS was previously attributed to the formation of neutral aggregates from polar interactions between the analyte functional groups and the nitrile from MeCN.^36^ Hence, the overall signal suppression—observed here—can be attributed to the same phenomenon. The extent of suppression among small molecules may also be attributed to differences in the surface affinity between peptides and amino acids. Peptides—particularly those with hydrophobic residues, exhibit greater surface affinity, which facilitates their enrichment at the droplet surface during the ESI process. This increases their probability of protonation and ion release even in the presence of MeCN vapor. Conversely, amino acids—being smaller and more polar than peptides—have greater tendency to reside within the droplet interior and are more susceptible to signal suppression via neutral aggregate formation or inefficient desolvation.

Among the vapors introduced into the nanoESI plume, PA exhibited the highest enhancement for both amino acids and peptides (Figure 2, and Tables S4 and S6). Notably, exposure to PA vapor resulted in *EF*s higher than those of AA vapor. This observation is consistent with a previous report that demonstrated a positive correlation between signal enhancement and the carbon chain length and boiling point of carboxylic acids.^10^ Among the organic solvents, vapors of EtOH and IPA also enhanced amino acid and peptides signals. This is in agreement with the earlier findings concerning enhanced detection of peptides,^10^ proteins,^10^ and oligonucleotides^11^ when EtOH and IPA vapors were introduced into the electrospray plume. The observed increase in detection sensitivity was attributed to the ability of organic vapors to lower the energy barrier required for the evaporation of solvent from droplets during the ESI process.^10,11^

### Real Sample Analysis by NanoESI-MS with Sequential Vapor Introduction

FlexESI was further applied in real sample analysis. A honey sample was diluted (200×) and directly analyzed in a full scan (*m*/*z* 70–250) to evaluate the effects of different vapor additives on signal intensity. Several common compounds in honey were identified based on their *m*/*z* values in the mass spectra (Figures S8 and S9). Their identities were further confirmed by MS/MS (Figures S10–S17) and high resolution MS/MS analyses (Table S7). Interestingly, only 13 signals were detected without vapor exposure, while the use of vapor additives increased the number of detected signals to 17 (AA), 20 (EtOH), and 21 (IPA), indicating enhanced detection sensitivity and analyte coverage (Table S8). Compounds such as valine, phenylalanine, lysine, and histidine, were detected only upon exposure to vapor additives. Moreover, vapor exposure resulted in the enhancement of several *m*/*z* signals in honey compared to those detected without vapor (Figures S8 and S9, and Table S8). For instance, choline (*EF* ≈ 3) and proline (*EF* ≈ 2) showed increased signal intensities upon exposure to IPA and AA vapors, respectively (Figure S9). We also used the MRM mode for the targeted analysis of amino acids in honey sample. The vapor additives enhanced the signal intensities of amino acids by ∼2 to 4 fold, depending on the analyte (Figure S18). Here, the majority of amino acids was enhanced by EtOH and AA vapors, suggesting that vapor-induced enhancement is also influenced by the sample matrix composition.

### Online Acid Vapor Gradients for Protein Interrogation

The second FlexESI method enabled the stepwise enrichment of individual weak acid vapors into the nanoESI plume. Here, a 10 μM protein (ubiquitin or cytochrome *c*)—dissolved in 10% (v/v) aqueous methanol solution with 1 mM ammonium acetate—was exposed to controlled gradient vapors of PA, AA, and FA during nanoESI-MS analysis. Unlike conventional methods that saturate the nanoESI plume with a fixed vapor level, we regulated the level of acid vapor to monitor protein conformational changes characterized by the CSD within 1 min. Each acid vapor additive influenced the CSDs of proteins to a different extent, as reflected in the mass spectra and distinct EIC profiles of their charge states (Figures 3, S19, and S20). Initially, without vapor exposure, ubiquitin was predominantly in a folded state with *z*_av_ in the range of ∼6.2 to 6.8. Upon exposure to acid vapors, unfolding was induced to varying degrees depending on the acid strength (Figure S20A). PA—the weakest of the three acid vapors—induced ubiquitin unfolding at Stage 4 (duty cycle, 50–55%), where *z*_av_ increased to 7.6. In contrast, AA and FA—being stronger acid vapors—induced unfolding at earlier stages: Stage 2 (*z*_av_ = 7.3; duty cycle, 20–25%) for AA and Stage 1 (*z*_av_ = 8.2; duty cycle, 5–10%) for FA. From Stage 4 to Stage 7, AA exposure resulted in complete unfolding, with +11 as the highest intensity charge state and a final *z*_av_ of 10.2 (duty cycle, 95–100%). Similarly, FA induced complete unfolding from Stage 3 onward, with the highest intensity charge states being +11 (Stages 3–5) and +10 (Stages 6–7), with a final *z*_av_ of 9.4 at Stage 7. On the other hand, PA exposure resulted in a bimodal CSD from Stage 4 to Stage 7 (*z*_av_ = 7.3), with coexisting folded and unfolded charge states, indicating partial unfolding (Figures 3 and S20A).

**Figure 3:**
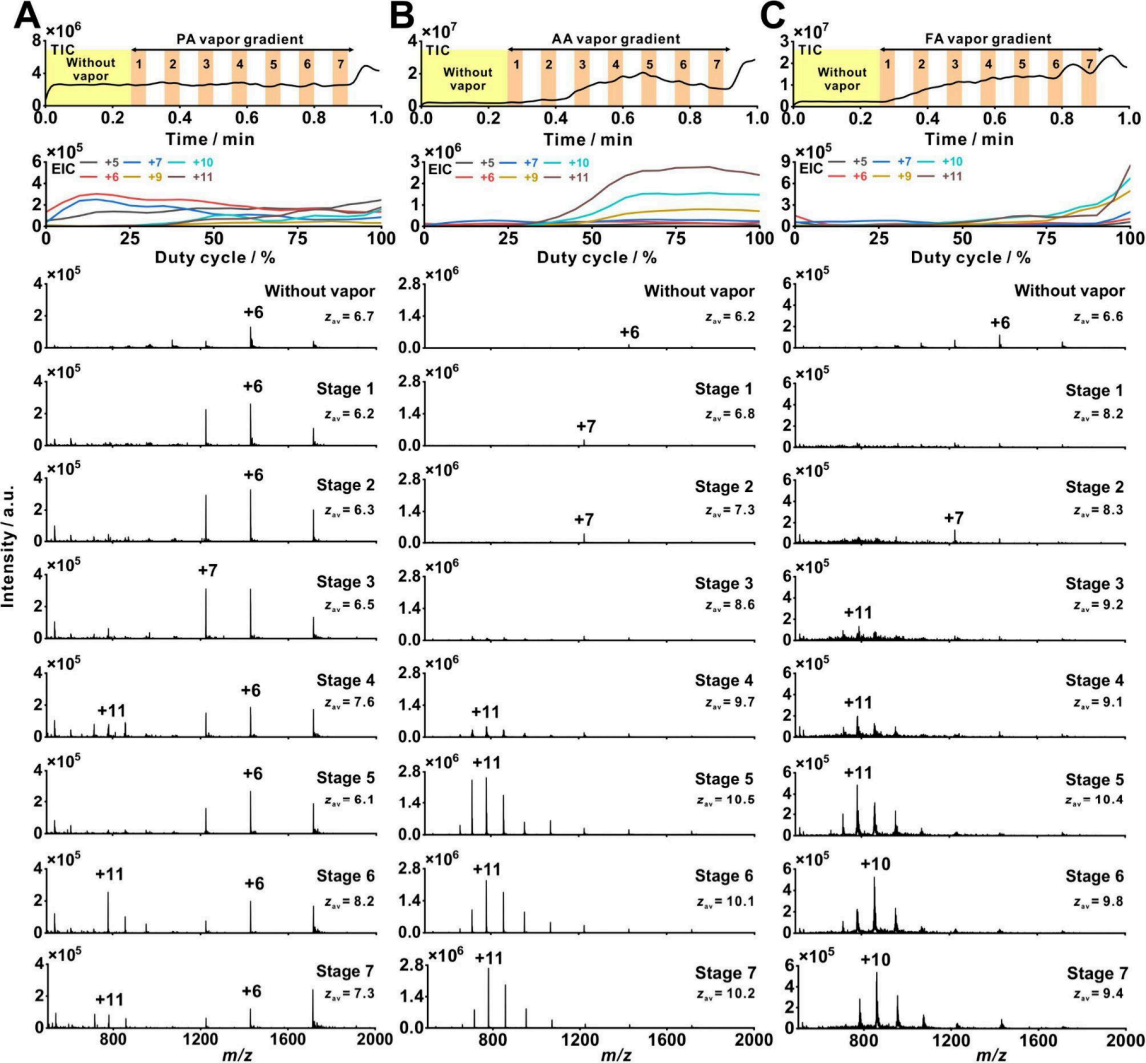
Effect of acid vapor gradients on CSD of ubiquitin. Shown are TICs and EICs of selected charge states (averaged; *n* = 3), and mass spectra corresponding to each stage (average of 5 data points) of the acid vapor ramp: (A) PA ramp; (B) AA ramp; (C) FA ramp. All acids were pure (∼99%) except 75% (v/v) aqueous FA solution. Sample solution: 10 μM ubiquitin dissolved in 10% (v/v) aqueous methanol solution with 1 mM ammonium acetate. Each stage corresponds to steps of the 40-s ramp—stage 1: steps 1–2, stage 2: steps 4–5, stage 3: steps 7–8, stage 4: steps 10–11, stage 5: steps 13–14, stage 6: steps 16–17, stage 7: steps 19–20 (for data processing details, see Supporting Information).

Apart from altering the CSD, the gradient vapor levels also influenced protein signal intensities. Upon exposure to increasing levels of PA vapor, the signal intensity of the folded charge state of ubiquitin (+6) increased at Stage 1 to 2, with intensities higher than the intensity without vapor—highlighting its potential for enhancing protein detection sensitivity (Figure 3). Similarly, as the vapor levels of AA and FA increased, the intensities of the unfolded ubiquitin charge states also increased. Among the tested acid vapors, AA produced the highest signal intensities of unfolded ubiquitin charge states. In addition to differences in acid strengths, this effect may be attributed to the gas-phase proton affinities of the acid vapors. AA vapor—with intermediate proton affinity relative to FA and PA, may represent a “sweet spot” in balancing efficient proton transfer with stable proton retention during ESI, leading to higher signal intensity compared to FA and PA under the same ESI conditions.

Temporal acid vapor gradients also affected the CSD and signal intensity of cytochrome *c* (Figures S19 and S20B). Introduction of acid vapors at Stage 1 already induced changes in the CSD of cytochrome *c*, even with PA vapor. Differences in acid strength influenced the unfolding behavior of cytochrome *c*. With PA vapor, *z*_av_ gradually increased and plateaued at Stage 5, while with AA and FA, the *z*_av_ plateaued early at Stage 3 (Figure S20B). Notably, cytochrome *c* underwent complete unfolding under increasing PA vapor levels (Figure S19). However, ubiquitin—with its compact structure and strong hydrogen bonding network^41,42^ —exhibited high structural stability and was only partially unfolded even at the highest PA vapor level (Stage 7; duty cycle, 95–100%; Figures 3 and S20A). Increasing acid vapor levels resulted in cytochrome *c* charge state intensities that were higher than the charge state intensities without vapor exposure, particularly in the case of AA. Gradient introduction of PA and FA vapors similarly enhanced the charge state signal intensities at specific stages of the ramp. AA vapor produced the highest cytochrome *c* charge state intensities, consistent with its effect on ubiquitin.

### Online Solvent Vapor Gradients for Protein Interrogation

In order to investigate the effect of increasing solvent vapor levels on protein ionization, we also conducted the individual stepwise enrichment of organic solvent vapors into the nanoESI plume. Using the same method for acid vapor gradients, increasing vapor levels of EtOH, IPA, and MeCN were gradually introduced to a 10 μM protein (ubiquitin or cytochrome *c*) dissolved in 10% (v/v) aqueous methanol solution with 1 mM ammonium acetate during nanoESI-MS analysis (Figures S21 and S22). A consistent, modest decrease in *z*_av_ was observed for both ubiquitin (Figures S21 and S23A) and cytochrome *c* (Figures S22 and S23B) upon exposure to increasing levels of EtOH and IPA vapors. This observation is in agreement with both findings of Hopper et al., which reported the effects of solvent vapor such as IPA, MeOH, MeCN on lowering myoglobin CSD,^43^ and of DeMuth et al., where they observed modest but consistent reduction of myoglobin *z*_av_ upon exposure to EtOH and IPA vapors.^31^ In our work, the extent of the effect of increasing levels of the organic vapors was clearly shown as distinct EIC profiles of the charge states and how they varied as the duty cycle was ramped up from 0 to 100% over 55 s. Additionally, the mass spectra at each stage of gradient vapor introduction revealed the impact of organic vapors on protein conformational stabilization. In the case of ubiquitin, the highest intensity charge state—without vapor exposure (*z*_av_ ≈ 6.6–6.9) and up to Stage 4 (*z*_av_ ≈ 5.6–6.0)—was +6. Upon exposure to higher levels of EtOH and IPA vapors (stages 5 to 7), the highest intensity charge state shifted from +6 to +5, with the +6 signal gradually decreasing until +5 became the highest intensity charge state (stage 7; *z*_av_ ≈ 5.2–5.4).

A similar trend was observed when cytochrome *c* was exposed to increasing levels of EtOH vapor. The dominant charge states initially included both +8 and +7. However, from Stage 2 onward, only the +7 charge state was detected, exhibiting the highest intensity. In the case of IPA vapor, a slight decrease in *z*_av_ was also observed in cytochrome *c* and both +8 and +7 charge states were still detectable at the final stage (Figures S22 and S23B). The gradual and consistent reduction in *z*_av_ for both proteins upon gradient vapor exposure may be attributed to the solvent vapor-assisted slowing of the desolvation process, which altered the ion evaporation dynamics and resulted in apparent stabilization of these proteins.^31^ In contrast, increasing levels of MeCN vapor did not induce significant conformational changes in either protein. Moreover, consistent with its effect on small molecules, the gradient introduction of MeCN vapor resulted in signal suppression for ubiquitin, while cytochrome *c* signal intensity remained unaffected.

### Coupling the Developed System with Liquid Chromatography

To further demonstrate the practical utility of FlexESI in analyses of complex samples, we coupled it with high-performance liquid chromatography (HPLC; see Supporting Information for details). Because FlexESI uses a much lower sample flow rate than the typical flow rates used in HPLC, the column effluent was split using a T-junction (Figure S24), and only a small fraction was directed to the ion source. The flow-splitting step can be skipped when using nanoflow liquid chromatography. Eight amino acids were separated on a hydrophilic interaction liquid chromatography column following a modified method described elsewhere.^44^ In one run, PA vapor was supplied to the electrospray plume via the described manifold, while in another run, no vapor was applied. The *EF*s in the presence of PA vapor ranged from ∼2 to ∼7 (Figure S25). Although, in this example, one selected vapor was infused during the entire chromatographic run, it is imaginable to program the system to apply different vapors to the separated zones eluting at different times to benefit from specific analyte-vapor interactions.

## Conclusions

We have demonstrated a facile system for the enhancement of ESI-MS signals, in which vapor additives are sequentially introduced to a nanoESI source. The *EF*s ranged from ∼2 to 15. A set of electronically controlled pinch valves is used to alternate between different vapor additives. This allows for the selection of a specific type of vapor additive or screening of multiple additives, which can enhance MS signals of particular analytes. This approach is particularly beneficial for real sample analysis, where matrix effects impact the ionization efficiency. Furthermore, we have automated the gradient introduction of acid and solvent vapors for enhanced protein detection sensitivity and rapid characterization of protein conformational changes via CSD. Importantly, this level of structural insight was obtained using a facile system that enables fast and controlled modulation of vapor levels. We believe that the proposed approach offers a practical and valuable tool for researchers in expanding nanoESI-MS applications, particularly in method development and in studying or modulating protein conformation.

## Supplementary Material



## Notes

Additional experimental details (liquid chromatography mass spectrometry; data processing); additional results and discussion (evaluation of precision, and vapor and analyte carryover); additional figures (S1–S25; scheme of electronic system; system tubing connections; hydrodynamic pump installation diagram; system optimization; relationship between the applied pressure and flow rate for nanoESI setup; within-laboratory reproducibility; effect of vapor carryover on aspartic acid signal; TICs and mass spectra of honey sample; MS/MS scan mass spectra; EICs of targeted amino acids in honey; acid vapor ramp on cytochrome *c*, *z*_av_ during acid vapor ramp; solvent vapor ramp on ubiquitin and cytochrome *c*; *z*_av_ during solvent vapor ramp; schematic of the LC coupling with a house-built nanoESI interface; EICs obtained during separation of amino acid followed by ionization with and without propionic acid vapor); additional tables (S1–S8; MRM transitions of amino acids and peptides; repeatability; within-laboratory reproducibility; *EF*s for amino acids; evaluation of vapor carryover; *EF*s for peptides; honey sample identification; enhanced *m*/*z* signals in honey); computer codes (PDF)
