# Supplementary material for: FlexESI: An Automated Vapor-Switching Interface for Enhanced Flexibility and Sensitivity in Electrospray Ionization
Source: ACS Meas Sci Au. 2025 Oct 8;5(6):814–22. doi: 10.1021/acsmeasuresciau.5c00086 (PMC12715638; doi:10.1021/acsmeasuresciau.5c00086)
Supplement: Supplementary file 1 [file tg5c00086_si_001.pdf]

SUPPORTING INFORMATION

FlexESI: An Automated Vapor-Switching Interface for Enhanced Flexibility and Sensitivity in Electrospray Ionization

Ying-Rong Hwang, Decibel P. Elpa, Pawel L. Urban*

Department of Chemistry, National Tsing Hua University

101, Section 2, Kuang-Fu Rd., Hsinchu, 300044, Taiwan

* Corresponding author:

P.L. Urban (urban@mx.nthu.edu.tw)

TABLE OF CONTENTS

Additional experimental details	S-3
Additional results and discussion	S-5
Additional figures	S-6
Additional tables	S-31
Computer codes	S-40

ADDITIONAL EXPERIMENTAL DETAILS

Liquid chromatography mass spectrometry

Liquid chromatography (LC) experiments were performed on a Hitachi Chromaster HPLC system (Hitachi, Tokyo, Japan). The amino acid separation was achieved on a hydrophilic interaction liquid chromatography (HILIC) column (SeQuant ZIC-HILIC; 150 mm × 2.1 mm, 3.5 µm particle size; Merck). A guard column (SeQuant ZIC-HILIC; 20 mm × 2.1 mm; Merck) was installed in front of the analytical column. The mobile phase composition and the gradient program were modified based on those described previously.[Shaba, C.; Urban, P. L. Metabolic Remodeling during Fructification of Enoki Mushroom. *Food Chem.* **2025**, *486*, 144613.] The mobile phases consisted of component A (0.1% formic acid (v/v) in acetonitrile) and component B (0.1% formic acid (v/v) in water). Gradient elution was programmed as follows: 0–15 min, 80–70% A; 15–15.1 min, 70–35% A; 15.1–35 min, 35–22% A; 35–35.1 min, 22–80% A; 35.1–50 min, 80% A. A flow rate of 0.2 mL min^{-1} was applied for chromatographic separation during the first 35 min, and then increased to 0.4 mL min^{-1} for re-equilibration until 50 min. In the final 10 min, the flow rate was reduced to 0.2 mL min^{-1} to re-establish the initial conditions. The column oven was set at 35 °C. The sample injection volume was 2.5 µL.

The commercial LC system was interfaced with the house-built nanoESI source via a PEEK T-junction splitter (thru-hole, 0.02 in; part no. P-727; IDEX Health & Science). A detailed schematic of the setup can be found in **Figure S24**. The flow split ratio was controlled by the inner diameter and length of the capillaries connected to the two outlets of the T-junction. One port was connected to a fused-silica capillary (length, 17.2 cm; ID, 0.075 mm; OD, 0.38 mm; part no. 1010-31945; GL Sciences) leading to waste, while the other port was connected to a capillary (length, 6 cm; ID, 0.02 mm; OD, 0.38 mm; part no. 1010-31442; GL Science). This capillary was further joined through a conductive micro union (part no. M-572; IDEX Health & Science) to an additional 3-cm capillary section (ID, 0.02 mm; OD, 0.38 mm; part no. 1010-31442; GL Sciences) before entering the house-built nanoESI emitter (length, 6 cm; ID, 20 µm; OD, 380 µm; part no. 1010-31442; GL Sciences). Both capillaries were fitted with a 1/16-inch PTFE tubing (length, 1.5 cm; ID, 0.3 mm; OD, 1.6 mm; Supelco) to secure the connection. Under these conditions, the LC effluent at an initial flow of 0.2 mL min^{-1} was reduced to ~ 1.2 µL min^{-1}. This flow rate was estimated using 50% (v/v) aqueous acetonitrile solution. The reduced flow was subsequently directed to a conductive micro union, maintained at a potential of +3.7 kV. The gas pressure of the modifier channel was set at 40 mbar (corresponding to ~ 132 mL min^{-1}).

Data processing

The extracted ion currents (EICs) for the selected ions were exported as ASCII files using LabSolutions software (version 5.97; Shimadzu). For amino acids and peptides, the ASCII files were then imported into MATLAB (version R2023b; MathWorks, Natick, MA) to determine the average signal intensity and standard deviation. The average signal intensity was determined from the last 6 s of the gas introduction period (20 s). The *EF* average and standard deviation were calculated using OriginPro (version 2024b; OriginLab, Northampton, MA, USA).

For data processing of proteins, the EICs of specific charge states were exported as ASCII files and then imported to MATLAB for average charge state (z_{av}) calculation using the following formula:

$$z_{av} = \frac{\sum_i^N z_i W_i}{\sum_i^N W_i} \qquad \text{(eq. S1)}$$

where W_i is the signal intensity of the ith charge state, and N is the number of observed analyte charge states in a given mass spectrum, and z_i is the net charge of the ith charge state. The data point averages were calculated using Excel software (version 2505; Microsoft Corporation, Redmond, WA, USA). OriginPro (version 2024b, OriginLab, Northampton, MA, USA) was used for data smoothing (adjacent-averaging; window, 2 points) and plotting. The protein mass spectrum was averaged, without vapor from 0-0.242 min and at each stage of the ramp as follows: stage 1: steps 1-2 from 0.267-0.300 min; stage 2: steps 4-5 from 0.367-0.400 min; stage 3: steps 7-8 from 0.467-0.500 min; stage 4: steps 10-11 from 0.567-0.600 min; stage 5: steps 13-14 from 0.667-0.700 min; stage 6: steps 16-17 from 0.767-0.800 min; and stage 7: steps 19-20 from 0.867-0.900 min.

ADDITIONAL RESULTS AND DISCUSSION

Evaluation of precision, and vapor and analyte carryover

The precision of amino acid nanoESI-MS analysis with sequential vapor introduction was evaluated. Ten consecutive measurements of 5 µM amino acids showed acceptable repeatability for all analytes under five different vapor conditions, with relative standard deviations (RSDs) of *EF*s ranging from 3.6% to 16.2% (**Table S2**). The within-laboratory reproducibility was also evaluated. The RSDs of *EFs* ranged from 9.9% to 64.9% across all analytes and vapor conditions, with analytes exposed to EtOH vapor showing the highest RSDs (**Table S3**). The high inter-day variability is possibly due to inconsistencies in the geometric alignment between the nanoESI emitter and the MS inlet after each reassembly. Although the absolute *EF*s varied across days, the signal enhancement trends induced by the five vapors were generally consistent for all analytes on different days (**Table S3** and **Figure S6**).

The effect of vapor carryover was assessed by evaluating how a preceding vapor influenced the impact of a subsequent vapor (expressed as *%EF difference*) on amino acid signals (**Table S5** and **Figure S7**). Results showed that the influence of vapor carryover varied among amino acids. Residual EtOH and MeCN vapors caused ion suppression in amino acids exposed to IPA (*%EF difference*; -12.57% to 20.59%) and PA (*%EF difference*; -8.09% to −15.23%), respectively. PA carryover had a moderate effect on amino acids exposed to AA vapor (*%EF difference*; <±10%), except for histidine, which showed high signal enhancement (*%EF difference*; 17.43%). IPA residual vapor suppressed tyrosine and tryptophan signals during MeCN exposure (*%EF difference*; tyrosine, -11.22%; tryptophan, -11.86%) but enhanced those of aspartic acid, lysine, glutamic acid, and histidine (*%EF difference*; 15.62% to 35.39%). Nevertheless, the majority of *%EF differences* were < 20%. Only three exceeded 20%, caused by EtOH vapor carryover during histidine exposure to IPA, and IPA vapor carryover during histidine and lysine exposure to MeCN (**Table S5**). Analysis of the blank after three replicate analyses of amino acid mixture showed that only aspartic acid exhibited a detectable signal when exposed to IPA and AA, with a high signal observed under IPA vapor (**Figure 2A**). Nevertheless, the signal intensities of most amino acids in the blank were negligible compared to their intensities upon vapor exposure.

ADDITIONAL FIGURES

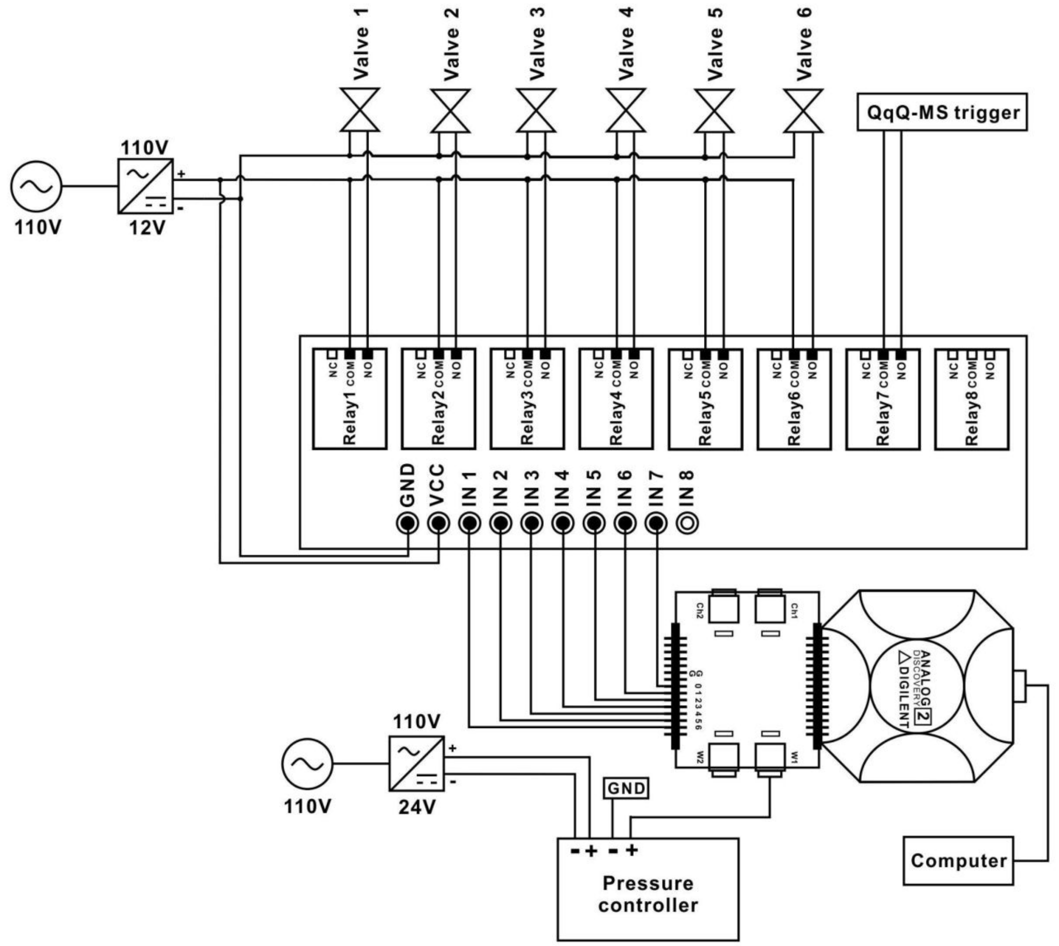

Figure S1. Scheme of the electronic system to control vapor introduction and trigger the mass spectrometer.

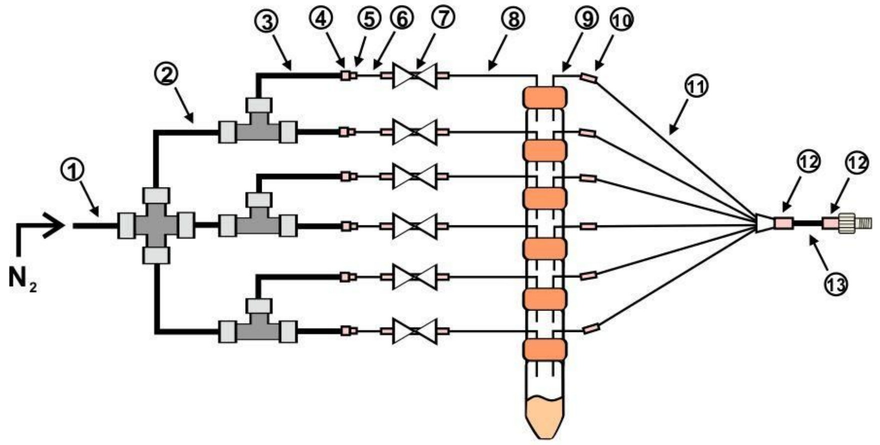

Figure S2. Tubing connections in the 6-channel vapor delivery system. (1) PTFE tubing (length, 3 cm, ID, 1.5 mm, OD, 3.2 mm); (2) PTFE tubing (length, 1 cm, ID, 1.5 mm, OD, 3.2 mm); (3) PTFE tubing (length, 10 cm, ID, 1.5 mm, OD, 3.2 mm); (4) silicone tubing (length, 1 cm, ID, 2 mm, OD, 5.0 mm); (5) silicone tubing (length, 0.8 cm, ID, 1.5 mm, OD, 2.5 mm); (6) PTFE tubing (ID, 0.8 mm, OD, 1.6 mm); (7) silicone tubing (length, 4 cm, ID, 0.8 mm, OD, 2.5 mm); (8) PTFE tubing (length, 19 cm, ID, 0.8 mm, OD, 1.6 mm); (9) PTFE tubing (length, 7 cm, ID, 0.8 mm, OD, 1.6 mm; (10) silicone tubing (length, 1 cm, ID, 0.8 mm, OD, 2.5 mm); (11) PTFE tubing (length, 28.5 cm, ID, 0.8 mm, OD, 1.6 mm); (12) silicone tubing (length, 1 cm, ID, 3 mm, OD, 5.0 mm); (13) PTFE tubing (length, 3 cm, ID, 1.5 mm, OD, 3.2 mm). Each tubing in the six channels is identical, except for tubing 6, which has been adjusted in length due to spatial arrangement.

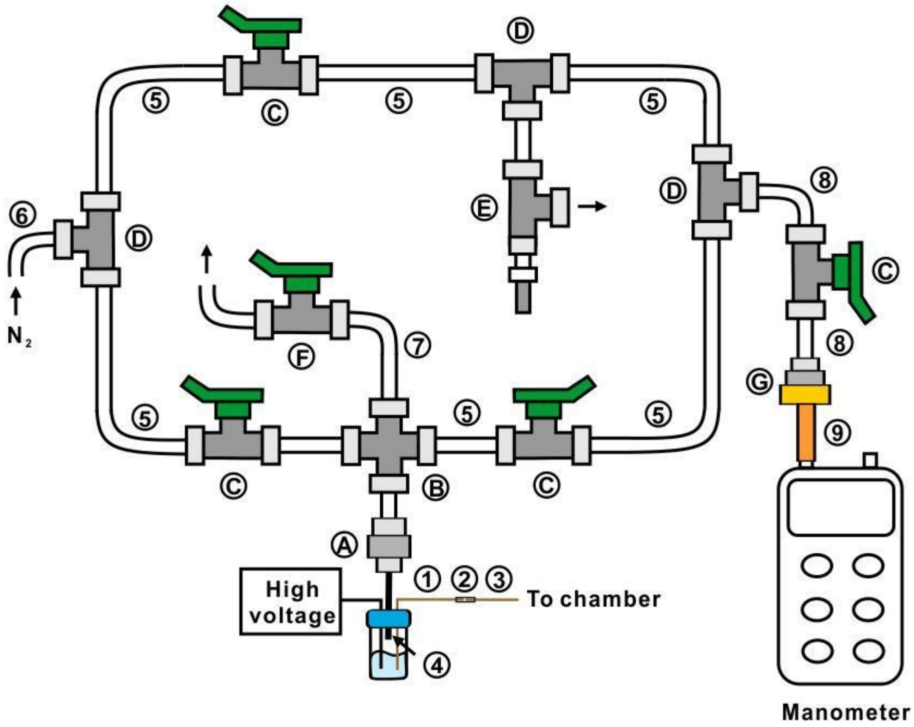

① Fused silica capillary, ID, 0.05 mm; OD, 0.375 mm; lenght, 600 mm
② PTFE tubing, ID, 0.38 mm; OD, 0.84 mm; length, 20 mm
③ Fused silica capillary, ID, 0.02 mm; OD, 0.375 mm; length, 60 mm
④ PTFE tubing, ID, 1.3 mm; OD, 1.8 mm; length, 32 mm
⑤ PTFE tubing, ID, 2.2 mm; OD, 3.2 mm; length, 208 mm
⑥ Stainless steel tubing, ID, 1.7 mm; OD, 3.2 mm; length, 37 mm
⑦ PTFE tubing, ID, 2.2 mm; OD, 3.2 mm; length, 58 mm
⑧ PTFE tubing, ID, 2.2 mm; OD, 3.2 mm; length, 125 mm
⑨ PU tubing, ID, 4.1 mm; OD, 5.9 mm; length, 113 mm

Ⓐ 1/8 to 1/16 inch, zero volume reducer union
Ⓑ 1/8 inch, union cross
Ⓒ 1/8 inch, plug valve
Ⓓ 1/8 inch, union tee
Ⓔ 1/8 inch, low flow angle pattern metering valve
Ⓕ 1/8 inch, toggle valve
Ⓖ 1/4 to 1/8 inch, union straight

Figure S3. Installation diagram of the hydrodynamic pump.

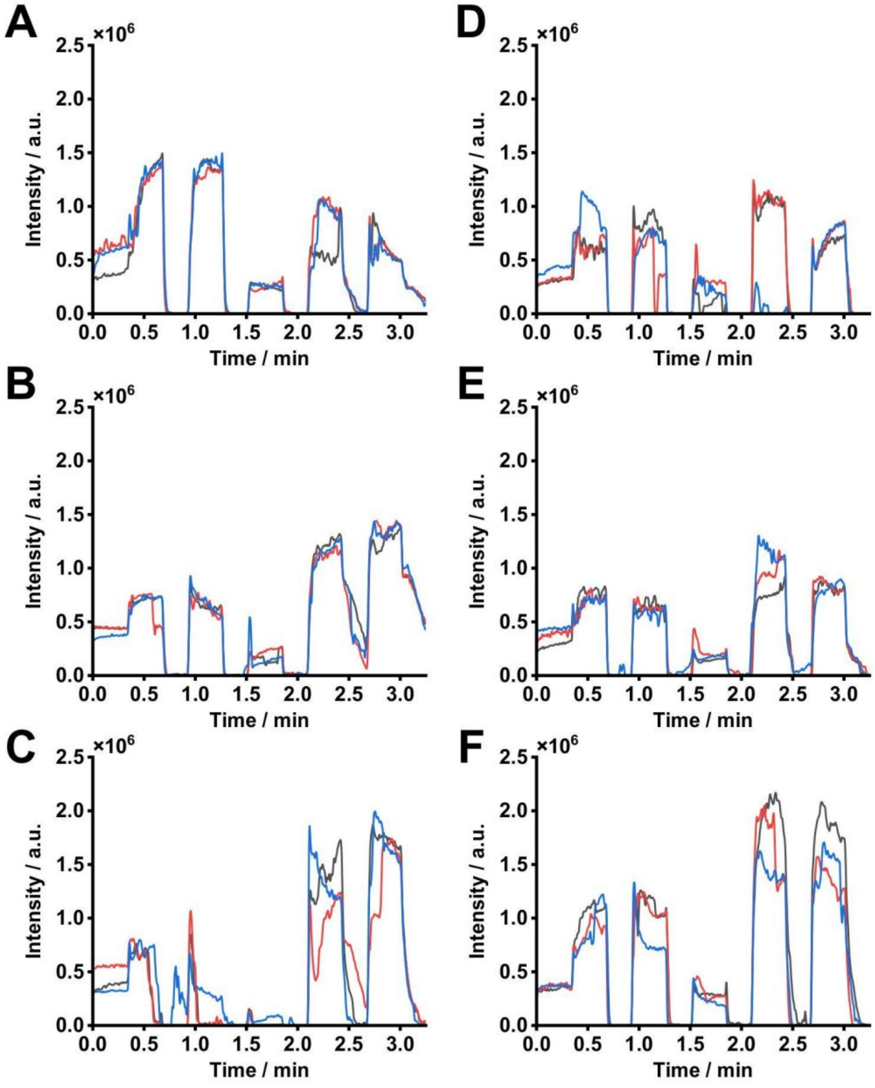

Figure S4. Optimization of the sequential vapor introduction system. Total ion currents (TICs) of amino acids exposed to sequentially introduced vapor additives. Each band corresponds to the vapor in sequence: EtOH–IPA–MeCN–PA–AA. Nitrogen pressure: (A) 80 mbar; (B) 120 mbar; and (C) 160 mbar under sample flow rate of 450 nL min^{-1}; sample solution flow rate: (D) 75 nL min^{-1}; (E) 100 nL min^{-1}; and (F) 129 nL min^{-1} under 80 mbar and 160 mbar nitrogen pressure for solvent and organic acid, respectively. Sample solution: 5 μM amino acid mixtures in 25% (v/v) aqueous methanol solution with 0.1% (v/v) AA. Replicates, n = 3.

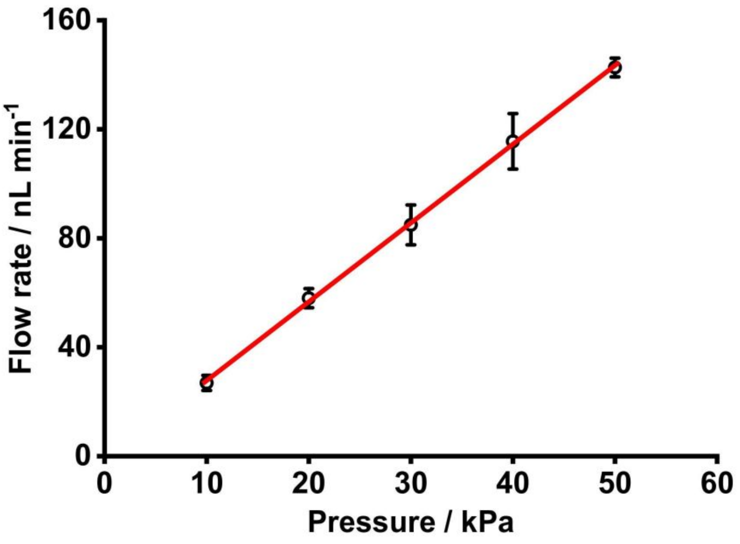

Figure S5. Relationship between flow rate and pressure applied to the nanoESI electrolyte solution vial. Sample solution used for calibration: 25% (v/v) aqueous methanol solution. Calibration equation: *Flow rate* = (2.89 ± 0.03) × *Pressure* + (-1.29 ± 0.99).

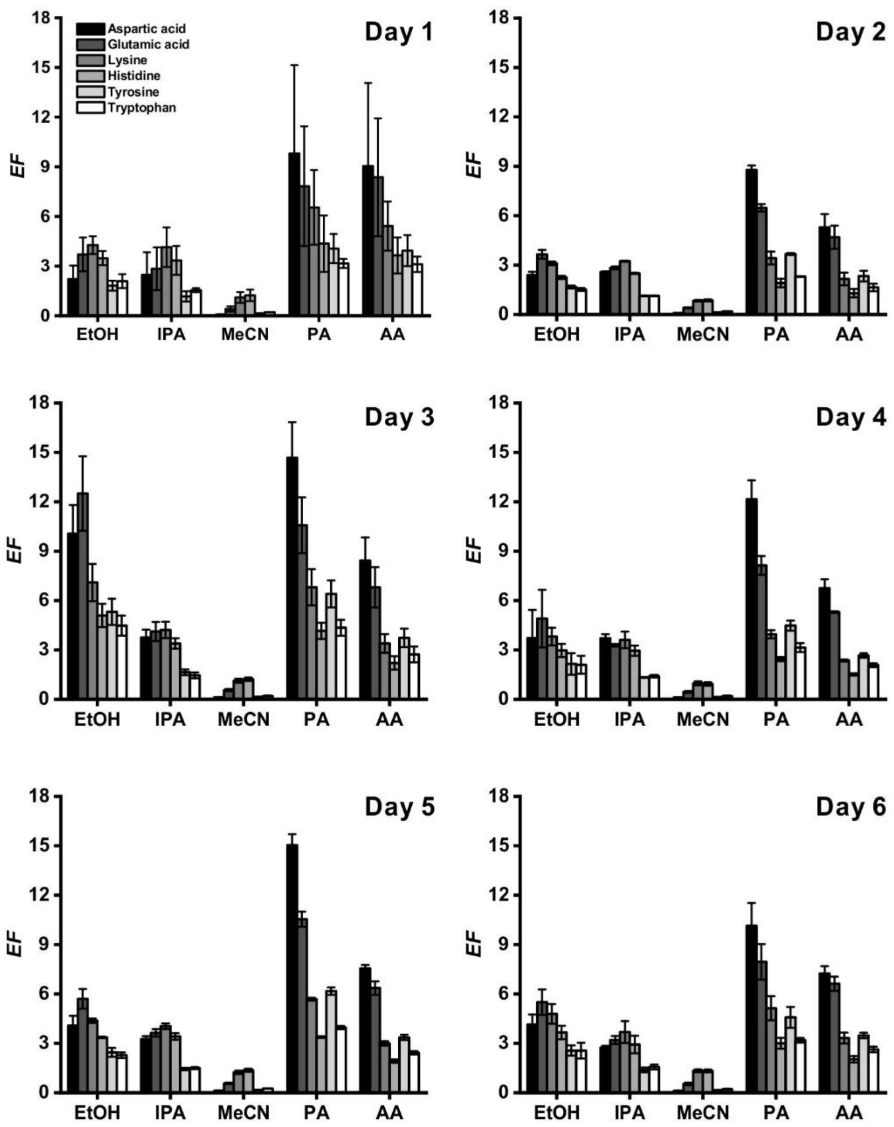

Figure S6. Reproducibility test (n = 6; 6 days; each day n = 3) for six amino acids sequentially exposed to vapor additives. Sample solution: 5 µM amino acid mixture in 25% (v/v) aqueous methanol solution with 0.1% (v/v) AA. *EF*s were calculated using the following equation: $EF = I/I_0$, where I_0 is the average signal intensity without vapor, while I is the average signal intensity with vapor.

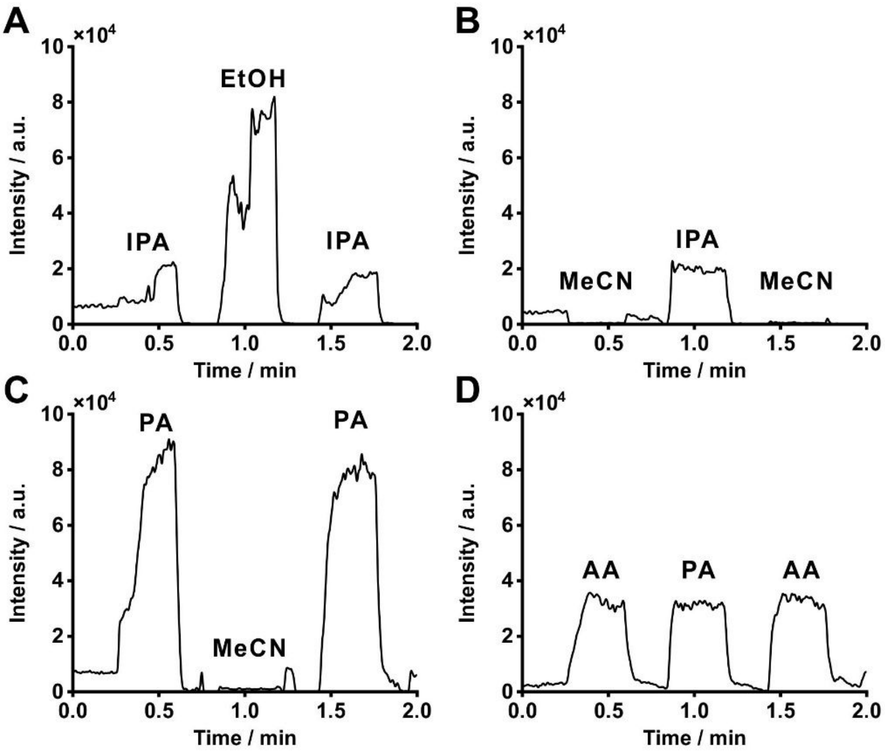

Figure S7. Assessment of vapor carryover by evaluating how prior vapor influences the impact of subsequent vapor on aspartic acid signal. The following vapor introduction sequences were tested: (A) IPA–EtOH–IPA; (B) MeCN–IPA–MeCN; (C) PA–MeCN–PA and (D) AA–PA–AA. Each vapor was sprayed for 20 s, followed by 15 s of nitrogen flushing in-between vapors to minimize residual interference. The carryover evaluation for other analytes is listed in **Table S3**. Sample solution: 5 µM amino acid mixture in 25% (v/v) aqueous methanol solution with 0.1% (v/v) AA. The aspartic acid EIC of one out of three replicates is shown.

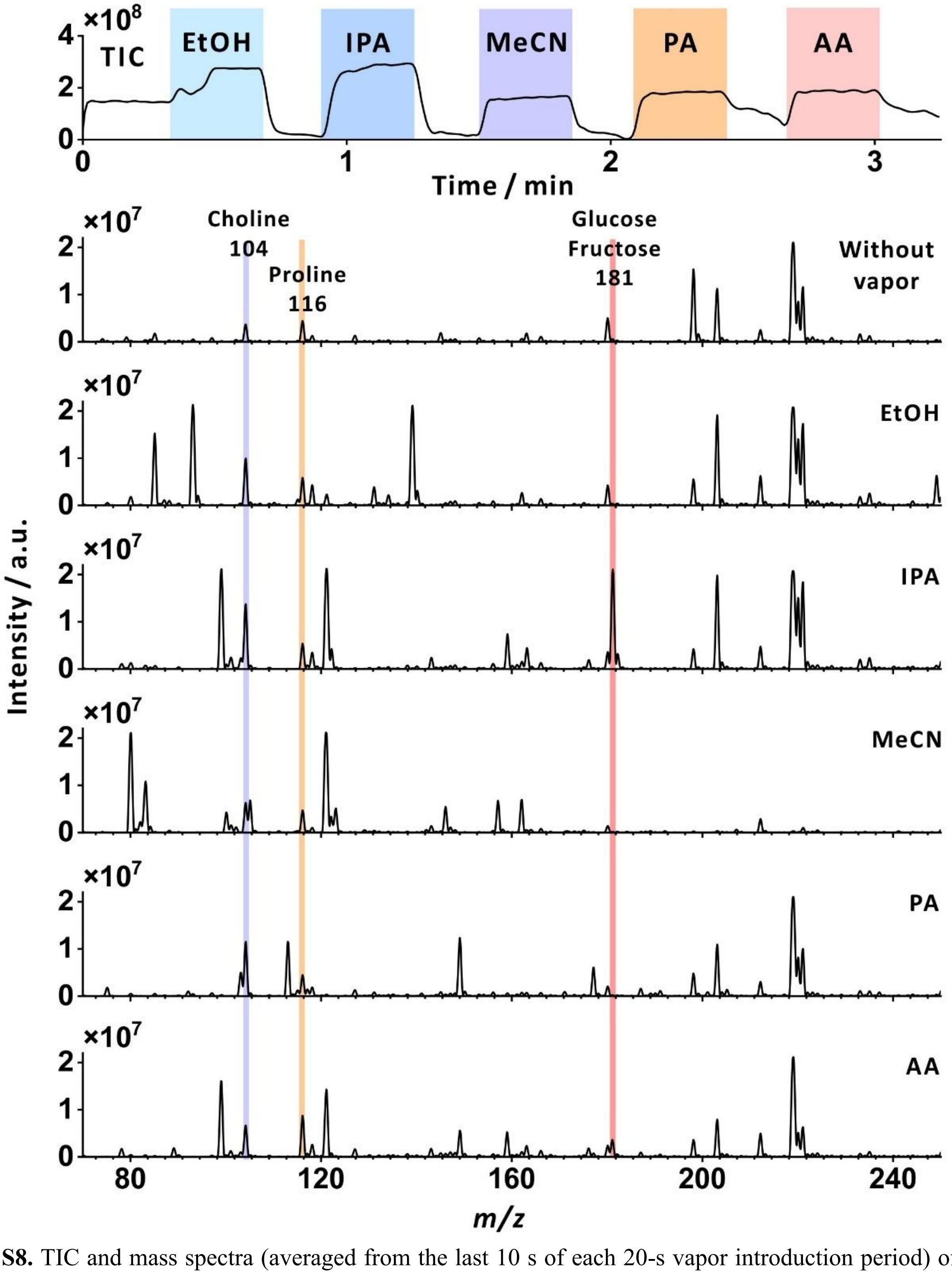

Figure S8. TIC and mass spectra (averaged from the last 10 s of each 20-s vapor introduction period) of honey sample exposed to sequentially introduced vapor additives analyzed in full scan mode (scan range: *m/z* 70–250). Sample solution: honey (200× dilution) in 25% (v/v) aqueous methanol solution with 0.1% (v/v) AA.

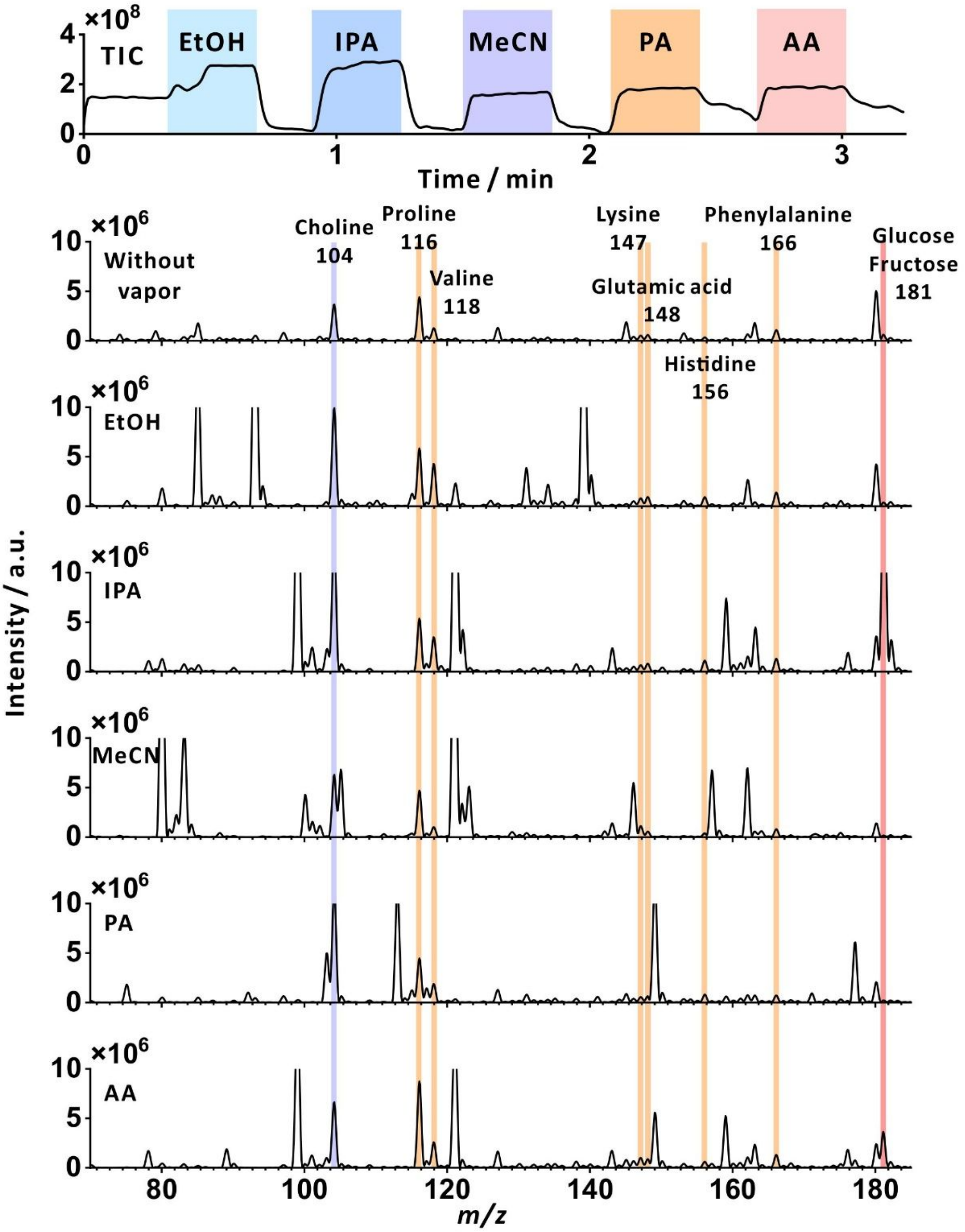

Figure S9. TIC and zoomed-in mass spectra of Figure S8. Sample solution: 200-fold diluted honey in 25% (v/v) aqueous methanol solution with 0.1% (v/v) AA.

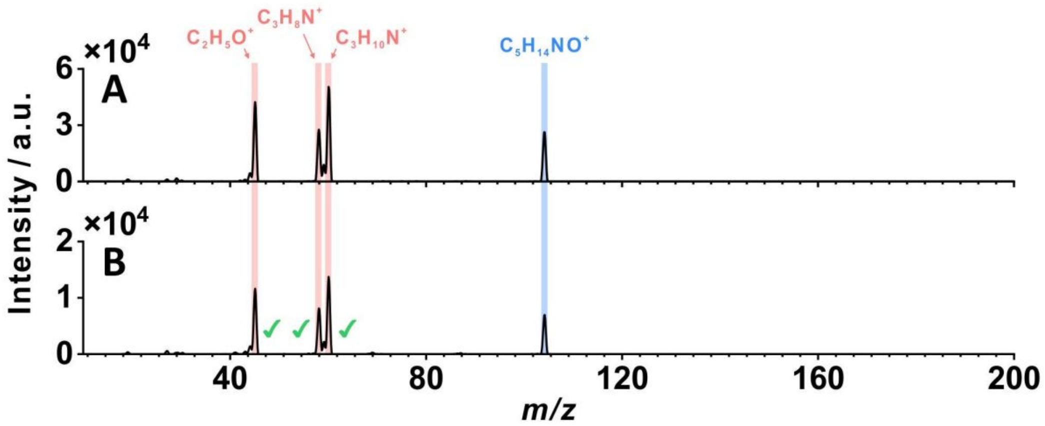

Figure S10. Mass spectra for the MS/MS analysis of choline in (A) the standard and (B) the honey sample. Collision voltage: -25 V. Standard solution: 5 μM choline in 25% (v/v) aqueous methanol solution with 0.1% (v/v) AA. Sample solution: honey (200× dilution) in 25% (v/v) aqueous methanol solution with 0.1% (v/v) AA.

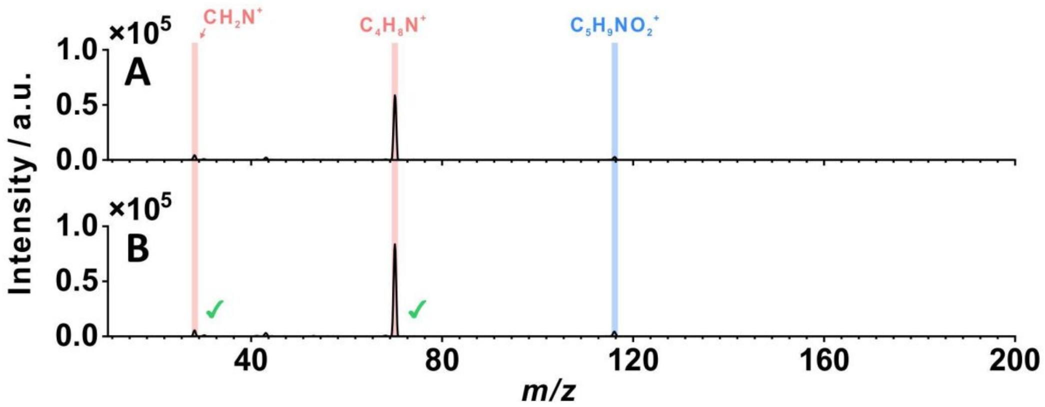

Figure S11. Mass spectra for the MS/MS analysis of proline in (A) the standard and (B) the honey sample. Collision voltage: -25 V. Standard solution: 5 μM proline in 25% (v/v) aqueous methanol solution with 0.1% (v/v) AA. Sample solution: honey (200× dilution) in 25% (v/v) aqueous methanol solution with 0.1% (v/v) AA.

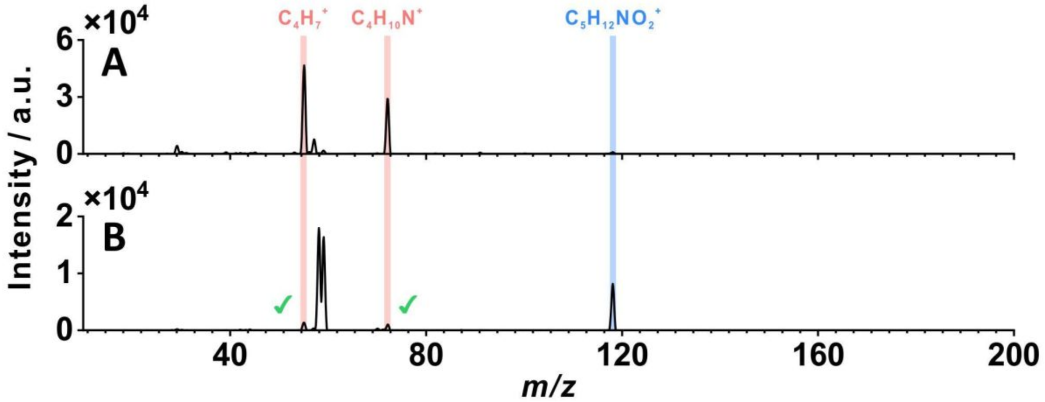

Figure S12. Mass spectra for the MS/MS analysis of valine in (A) the standard and (B) the honey sample. Collision voltage: -25 V. Standard solution: 5 μM valine in 25% (v/v) aqueous methanol solution with 0.1% (v/v) AA. Sample solution: honey (200× dilution) in 25% (v/v) aqueous methanol solution with 0.1% (v/v) AA.

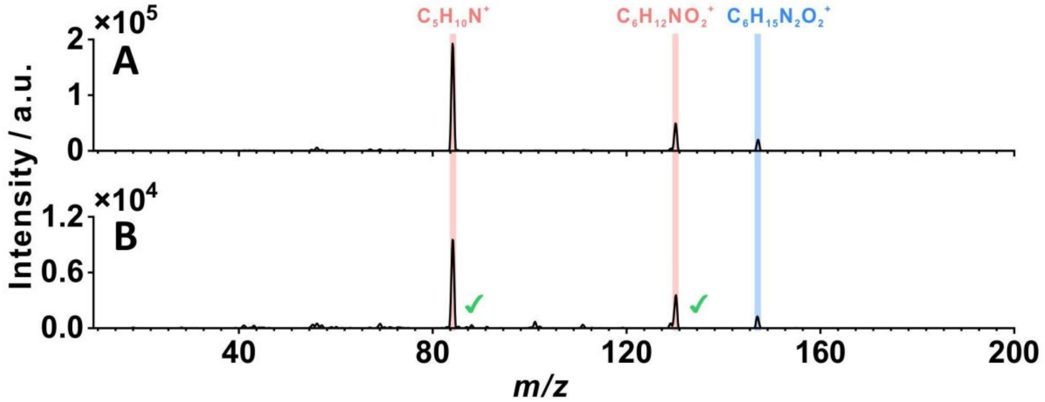

Figure S13. Mass spectra for the MS/MS analysis of lysine in (A) the standard and (B) the honey sample. Collision voltage: -18 V. Standard solution: 5 μM lysine in 25% (v/v) aqueous methanol solution with 0.1% (v/v) AA. Sample solution: honey (200× dilution) in 25% (v/v) aqueous methanol solution with 0.1% (v/v) AA.

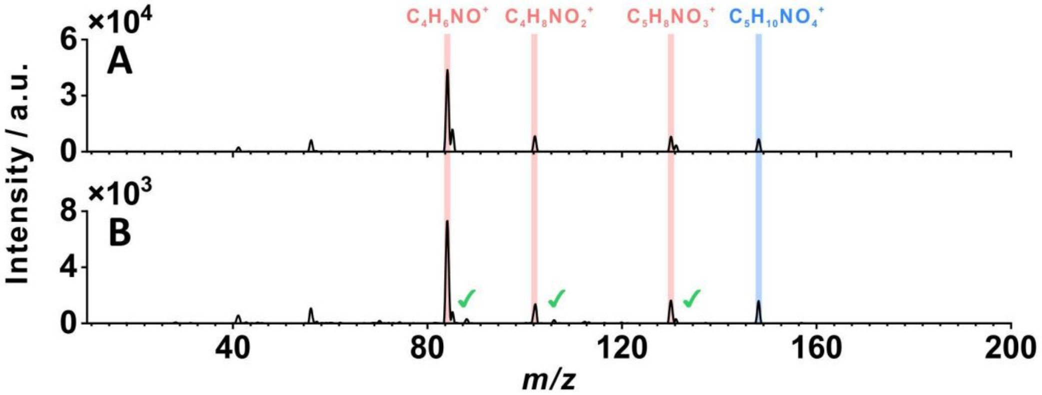

Figure S14. Mass spectra for the MS/MS analysis of glutamic acid in (A) the standard and (B) the honey sample. Collision voltage: -18 V. Standard solution: 5 μM glutamic acid in 25% (v/v) aqueous methanol solution with 0.1% (v/v) AA. Sample solution: honey (200× dilution) in 25% (v/v) aqueous methanol solution with 0.1% (v/v) AA.

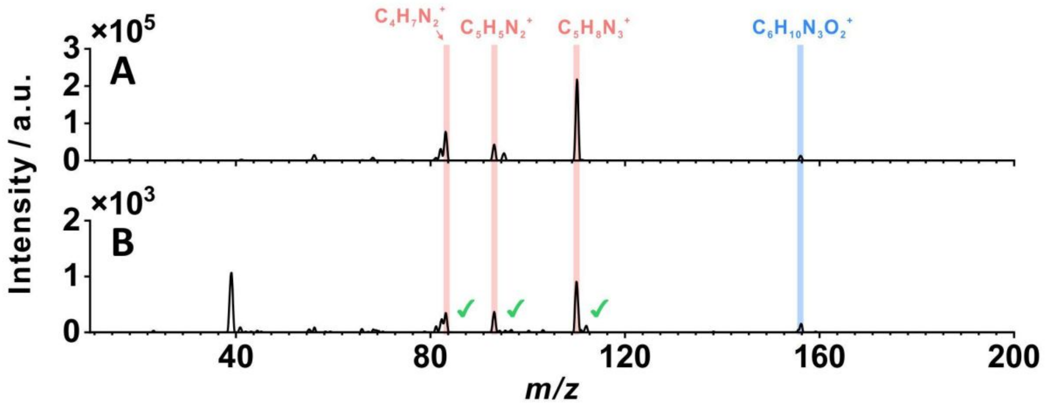

Figure S15. Mass spectra for the MS/MS analysis of histidine in (A) the standard and (B) the honey sample. Collision voltage: -22 V. Standard solution: 5 μM histidine in 25% (v/v) aqueous methanol solution with 0.1% (v/v) AA. Sample solution: honey (200× dilution) in 25% (v/v) aqueous methanol solution with 0.1% (v/v) AA.

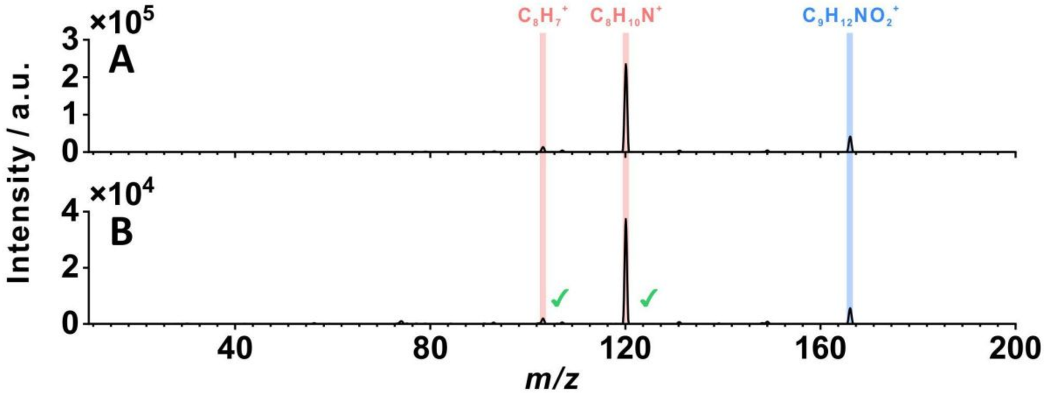

Figure S16. Mass spectra for the MS/MS analysis of phenylalanine in (A) the standard and (B) the honey sample. Collision voltage: -15 V. Standard solution: 5 μM phenylalanine in 25% (v/v) aqueous methanol solution with 0.1% (v/v) AA. Sample solution: honey (200× dilution) in 25% (v/v) aqueous methanol solution with 0.1% (v/v) AA.

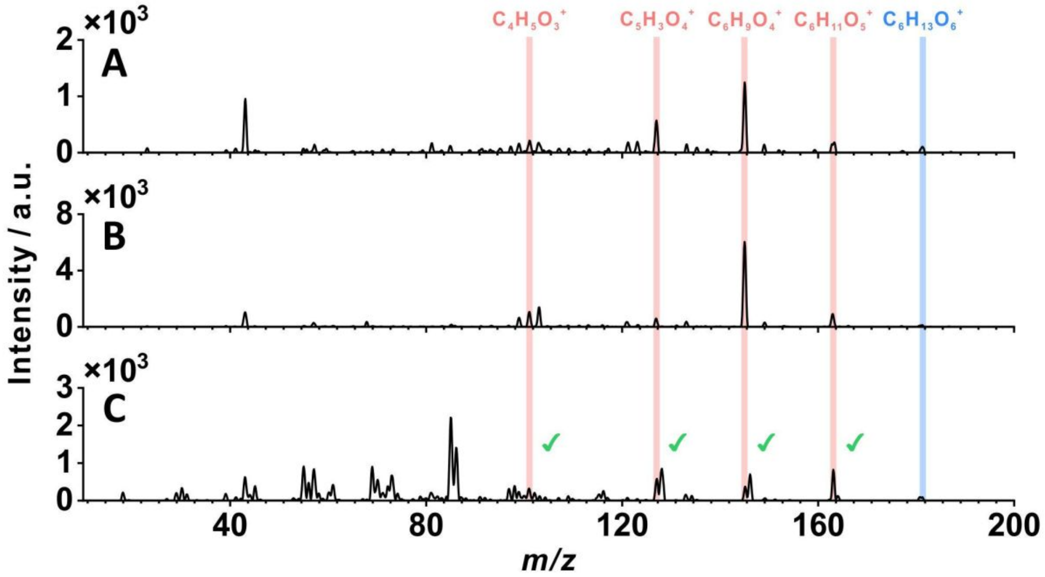

Figure S17. Mass spectra for the MS/MS analysis of glucose/fructose in (A) the glucose standard (B) the fructose standard and (C) honey sample. Collision voltage: -20 V. Standard solution: 5 μM glucose/fructose in 25% (v/v) aqueous methanol solution with 0.1% (v/v) AA. Sample solution: honey (200× dilution) in 25% (v/v) aqueous methanol solution with 0.1% (v/v) AA.

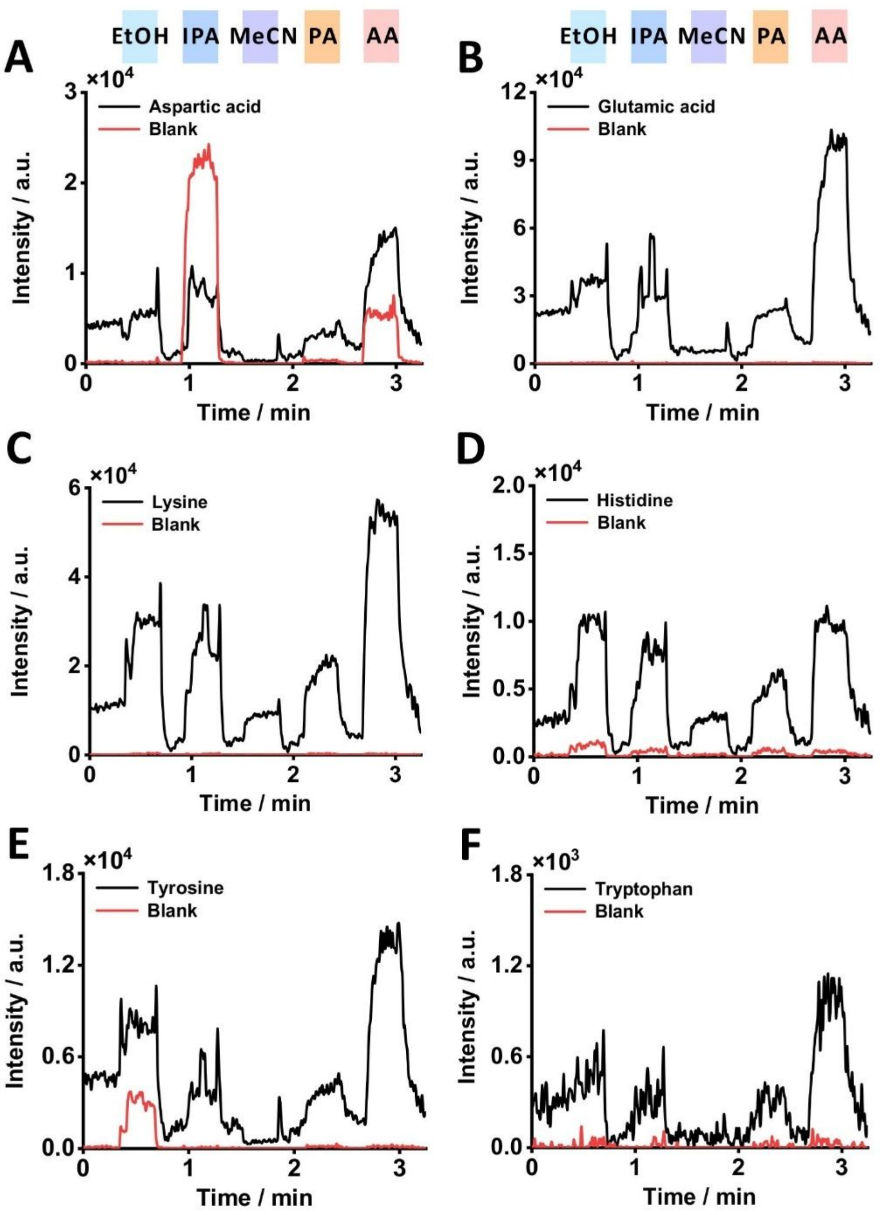

Figure S18. EICs of targeted amino acids in a honey sample exposed to sequentially introduced vapor additives. Vapor sequence: EtOH–IPA–MeCN–PA–AA: (A) aspartic acid; (B) glutamic acid; (C) lysine; (D) histidine; (E) tyrosine; (F) tryptophan. Sample solution: honey (200× dilution) in 25% (v/v) aqueous methanol solution with 0.1% (v/v) AA. One out of three replicates is shown.

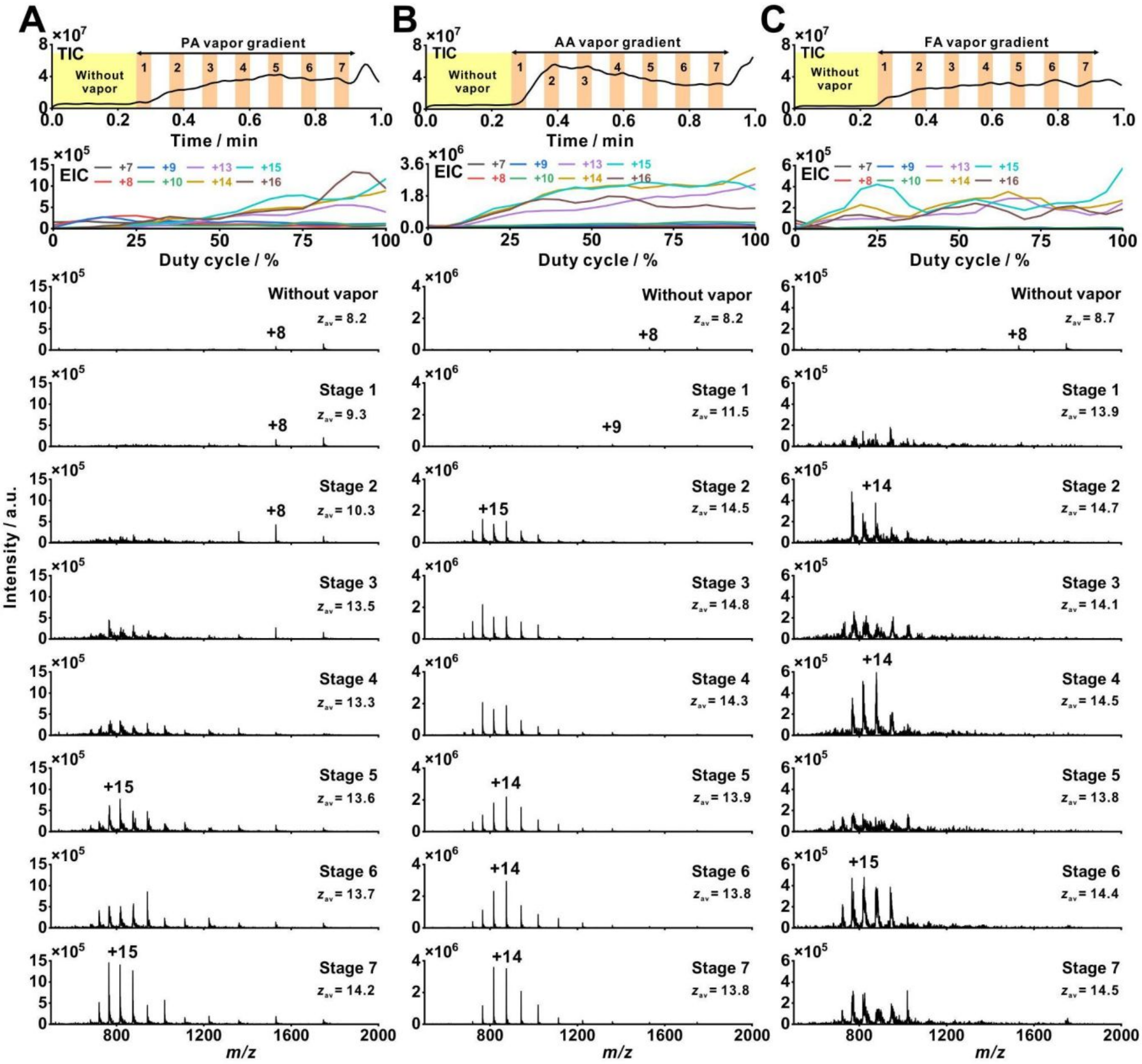

Figure S19. Effect of acid vapor gradients on CSD of cytochrome *c*. Shown are TICs and EICs of selected charge states (averaged; $n = 3$), and mass spectra corresponding to each stage (average of 5 data points) of the acid vapor ramp: (A) PA ramp; (B) AA ramp; (C) FA ramp. All acids were used pure (~99%) except 75% (v/v) aqueous FA solution. Sample solution: 10 µM cytochrome *c* dissolved in 10% (v/v) aqueous methanol solution with 1 mM ammonium acetate. Each stage corresponds to steps of the 40-s ramp—stage 1: steps 1-2, stage 2: steps 4-5, stage 3: steps 7-8, stage 4: steps 10-11, stage 5: steps 13-14, stage 6: steps 16-17, stage 7: steps 19-20 (for data processing details, see **Additional Experimental Details**).

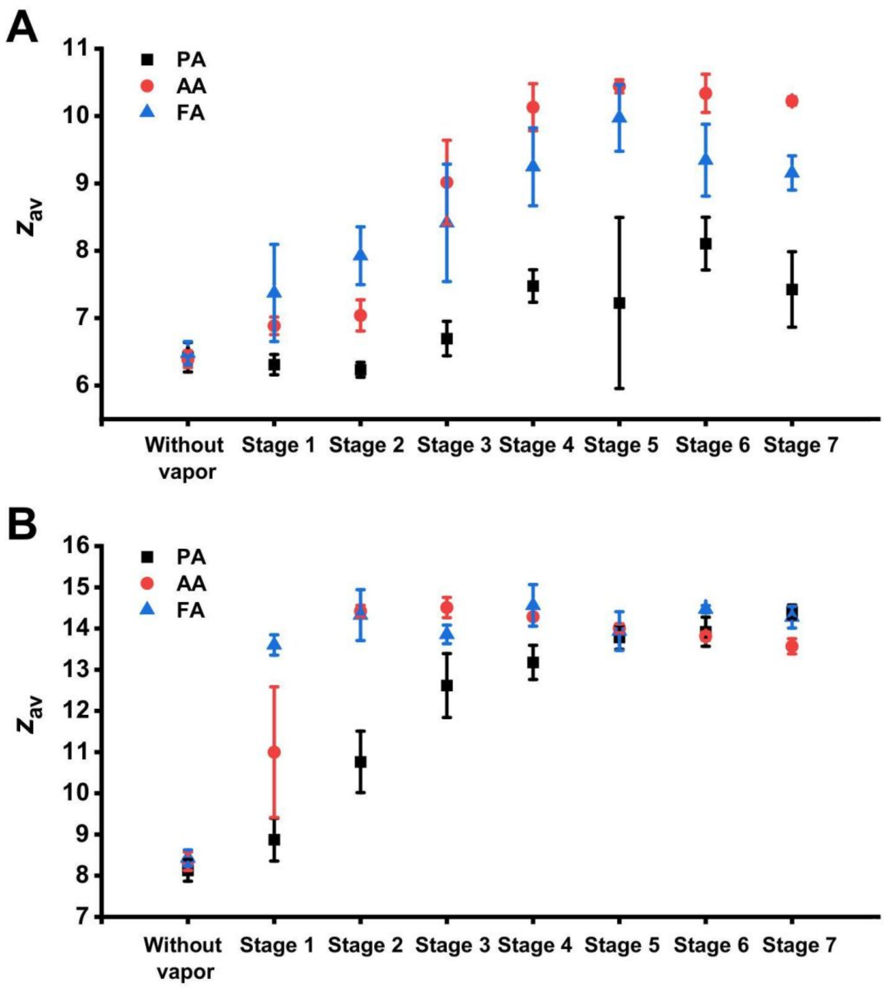

Figure S20. Average charge states of tested proteins without vapor additive and at each stage of the acid vapor ramp: (A) 10 μM ubiquitin dissolved in 10% (v/v) aqueous methanol solution with 1 mM ammonium acetate and (B) 10 μM cytochrome *c* dissolved in 10% (v/v) aqueous methanol solution with 1 mM ammonium acetate. Each data point represents the mean of the average charge state, with error bars indicating the standard deviation (n = 3). Acid vapor ramps: PA (black square), AA (red circle), and FA (blue triangle).

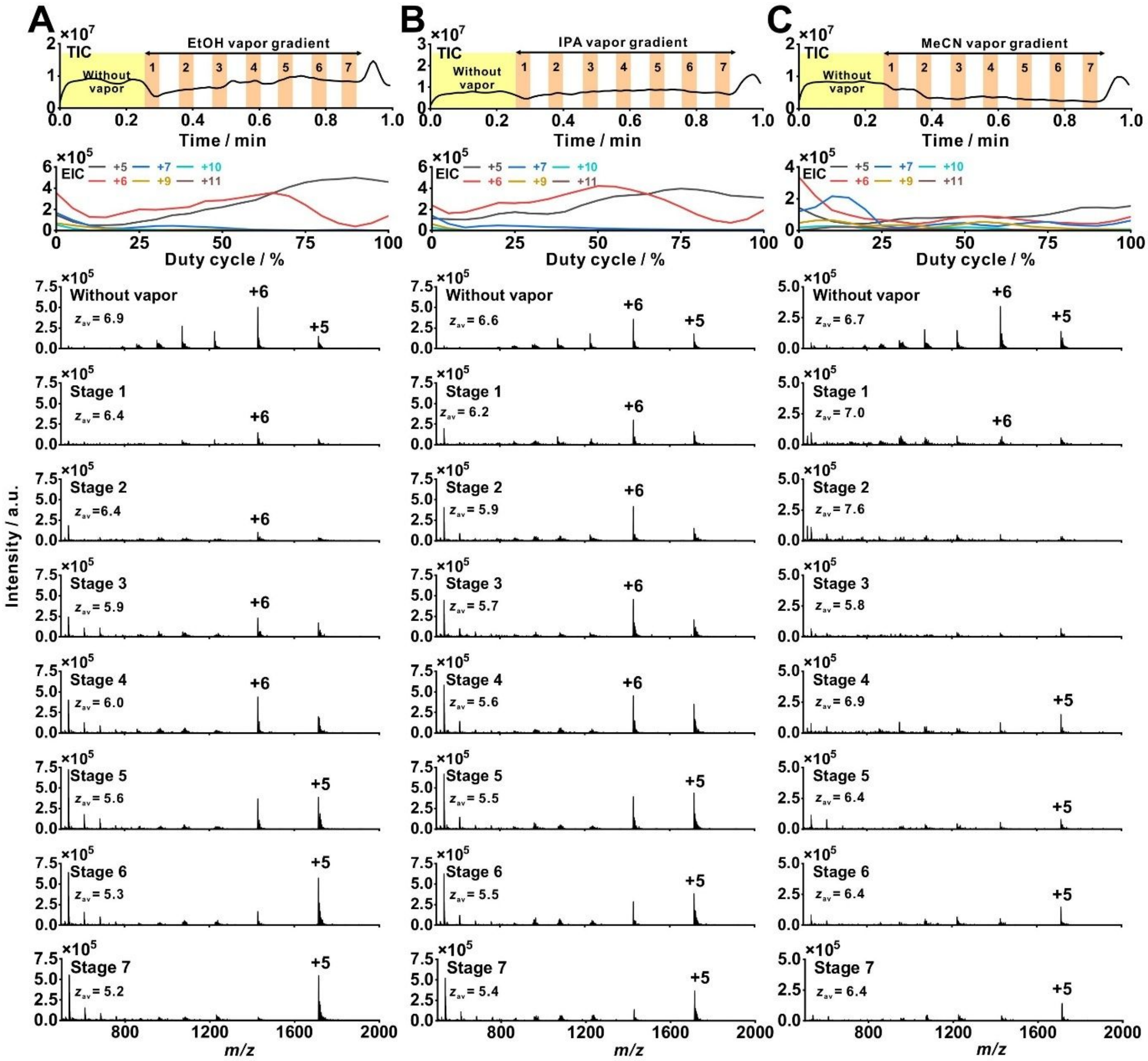

Figure S21. Effect of solvent vapor gradients on CSD of ubiquitin. Shown are TICs and EICs of selected charge states (averaged; n = 3), and mass spectra corresponding to each stage (average of 5 data points) of the solvent vapor ramp: (A) EtOH ramp; (B) IPA ramp; (C) MeCN ramp. All solvents were used pure (≥99.5%). Sample solution: 10 μM ubiquitin dissolved in 10% (v/v) aqueous methanol solution with 1 mM ammonium acetate. Each stage corresponds to steps of the 40-s ramp—stage 1: steps 1-2, stage 2: steps 4-5, stage 3: steps 7-8, stage 4: steps 10-11, stage 5: steps 13-14, stage 6: steps 16-17, stage 7: steps 19-20 (for data processing details, see **Additional Experimental Details**).

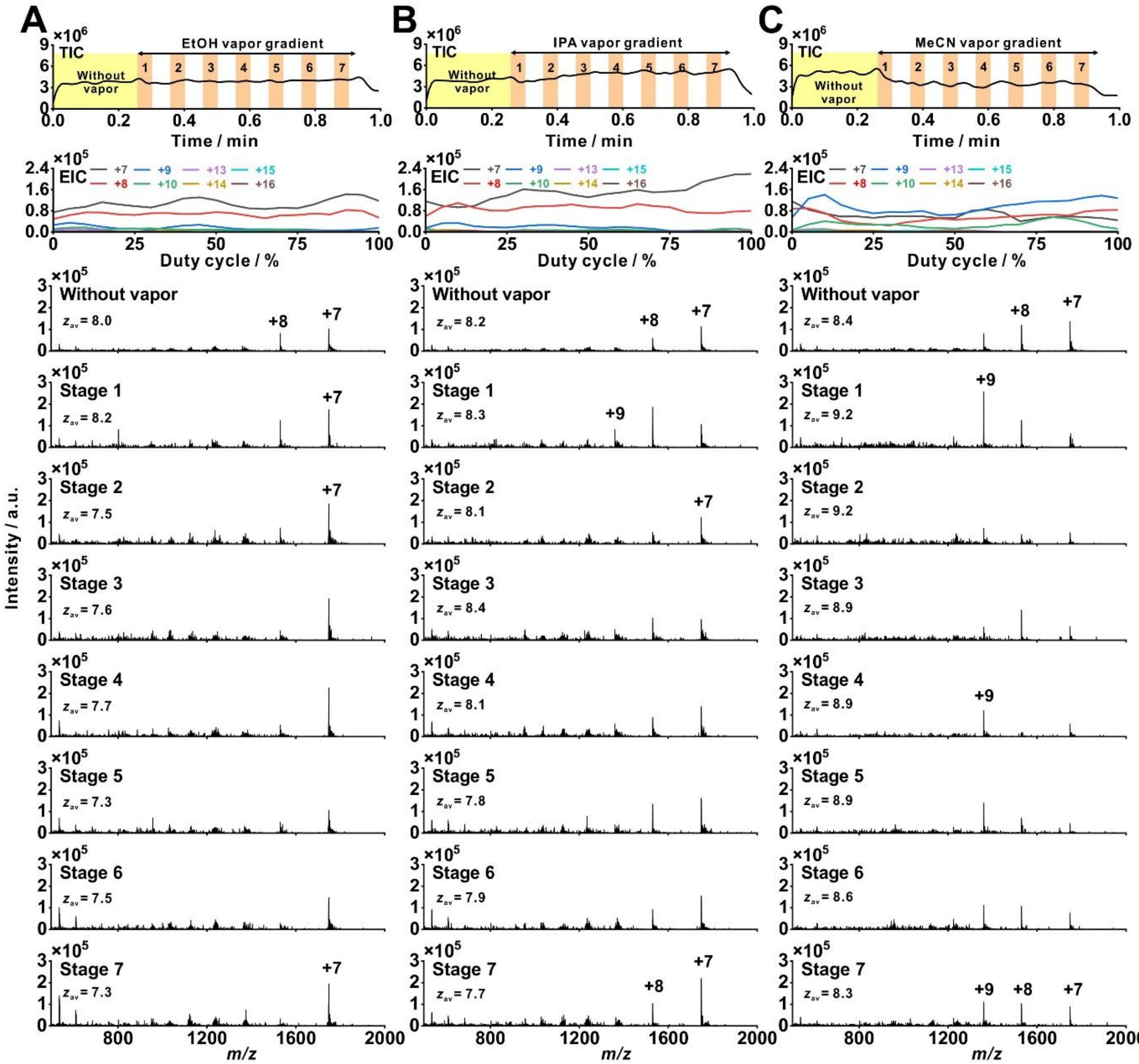

Figure S22. Effect of solvent vapor gradients on CSD of cytochrome *c*. Shown are TICs (averaged; $n = 3$), EICs of selected charge states (averaged; $n = 3$), and mass spectra corresponding to each stage (average of 5 data points) of the solvent vapor ramp: (A) EtOH ramp; (B) IPA ramp; (C) MeCN ramp. All solvents were used pure (≥99.5%). Sample solution: 10 μM cytochrome *c* dissolved in 10% (v/v) aqueous methanol solution with 1 mM ammonium acetate. Each stage corresponds to steps of the 40-s ramp—stage 1: steps 1-2, stage 2: steps 4-5, stage 3: steps 7-8, stage 4: steps 10-11, stage 5: steps 13-14, stage 6: steps 16-17, stage 7: steps 19-20 (for data processing details, see **Additional Experimental Details**).

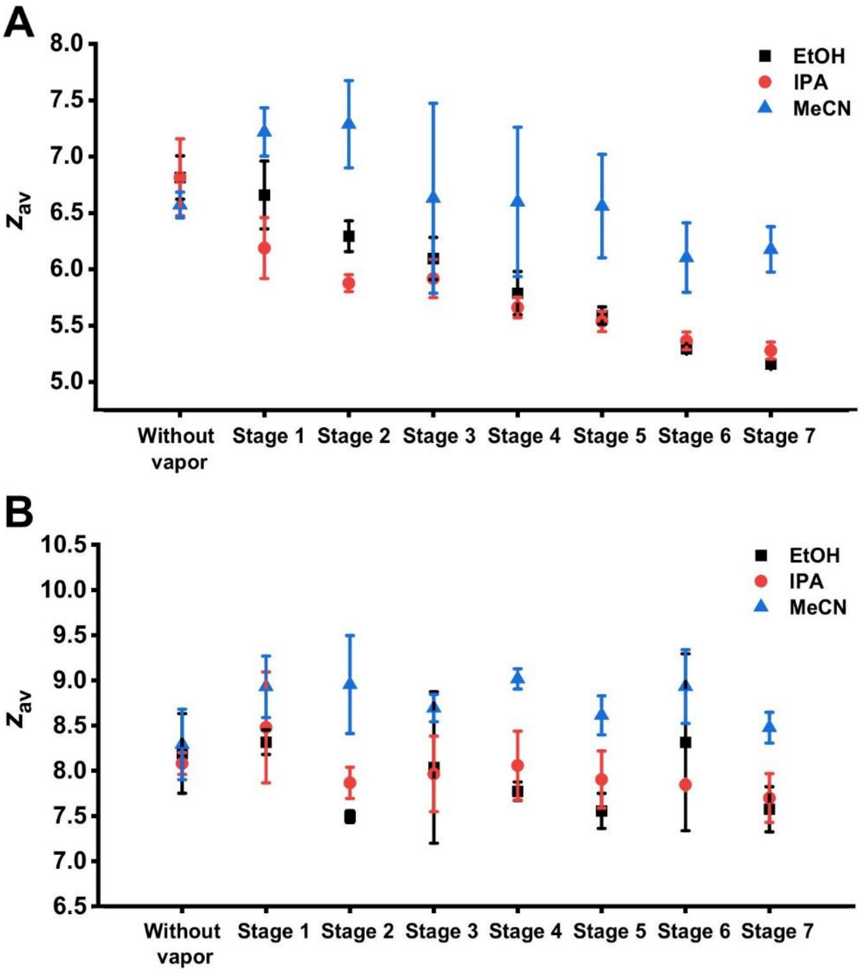

Figure S23. Average charge states of tested proteins without vapor additive and at each stage of the solvent vapor ramp: (A) 10 µM ubiquitin dissolved in 10% (v/v) aqueous methanol solution with 1 mM ammonium acetate and (B) 10 µM cytochrome *c* dissolved in 10% (v/v) aqueous methanol solution with 1 mM ammonium acetate. Each data point represents the mean of the average charge state, with error bars indicating the standard deviation (n = 3). Solvent vapor ramps: EtOH (black square), IPA (red circle), and MeCN (blue triangle).

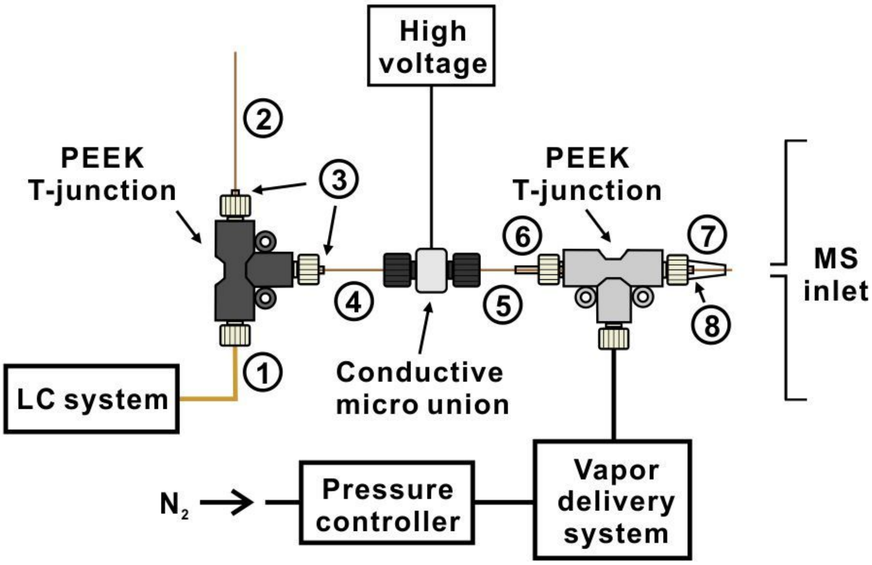

Figure S24. Schematic of the LC coupling with a house-built nanoESI interface: (1) PEEK tubing (length, 70 cm, ID, 0.13 mm, OD, 1.6 mm); (2) fused silica capillary (length, 17.2 cm, ID, 0.075 mm, OD, 0.375 mm); (3) PTFE tubing (length, 1.5 cm, ID, 0.3 mm, OD, 1.6 mm); (4) fused silica capillary (length, 6 cm, ID, 0.02 mm, OD, 0.375 mm); (5) fused silica capillary (length, 3 cm, ID, 0.02 mm, OD, 0.375 mm); (6) PTFE tubing (length, 3 cm, ID, 0.3 mm, OD, 1.6 mm); (7) fused silica capillary (length, 6 cm, ID, 0.02 mm, OD, 0.375 mm); (8) PTFE tubing (length, 2 cm, ID, 0.8 mm, OD, 1.6 mm).

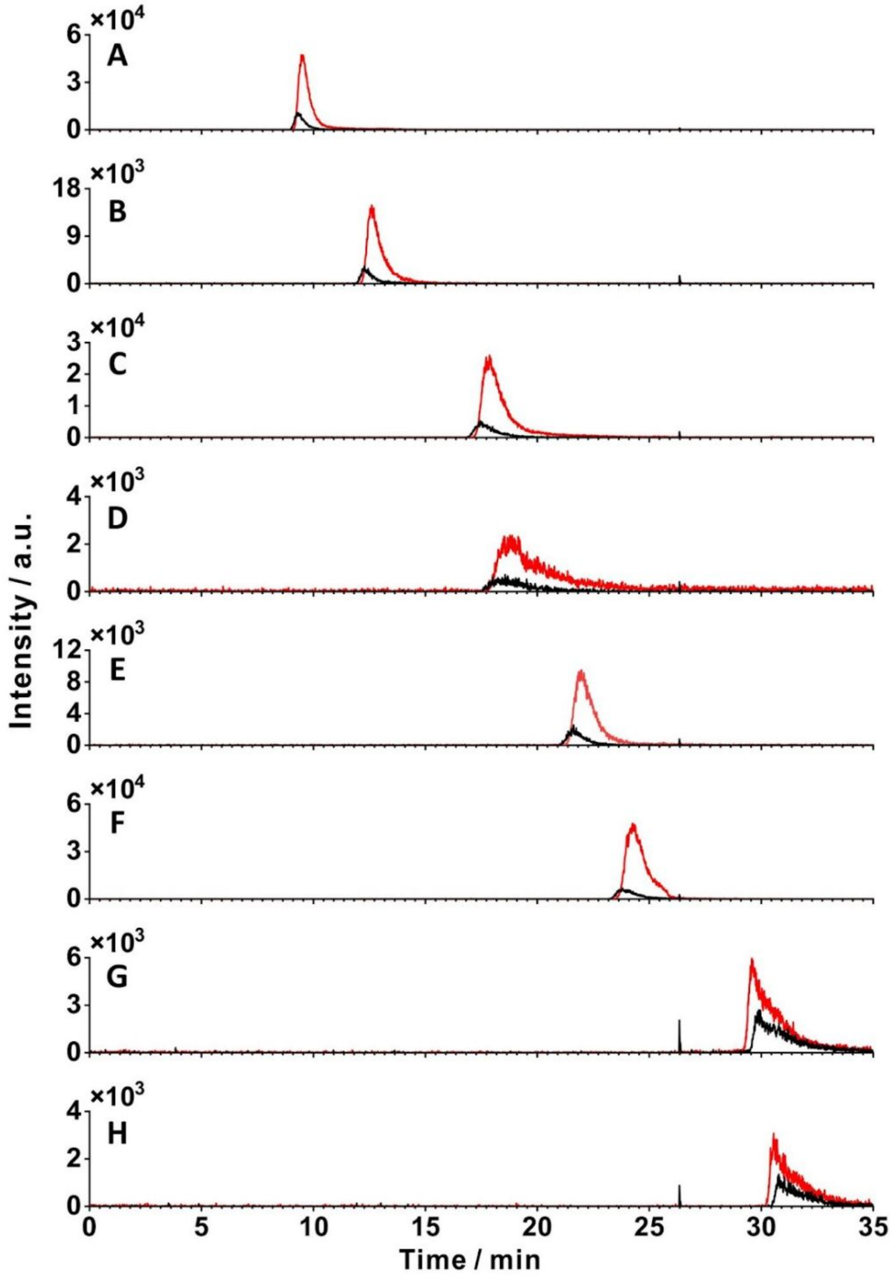

Figure S25. EICs obtained during separation of amino acid followed by ionization with and without propionic acid vapor: (A) tryptophan; (B) tyrosine; (C) glutamic acid; (D) aspartic acid; (E) serine; (F) citrulline; (G) histidine; (H) lysine. Sample solution: 10 µM amino acid mixture in 80% (v/v) aqueous acetonitrile solution with 0.1% (v/v) FA. The black line corresponds to the separation without vapor exposure, whereas the red line corresponds to the separation with vapor exposure.

ADDITIONAL TABLES

Table S1. MRM transitions of amino acids and peptides.

Analyte	Precursor ion *m/z*	Product ion *m/z*	Collision voltage / V
serine	106.1	60.1	-18
aspartic acid	134.1	73.9	-22
lysine	147.0	84.1	-25
glutamic acid	148.1	84.1	-20
histidine	156.1	110.1	-22
citrulline	176.1	159.1	-15
tyrosine	182.1	136.2	-20
tryptophan	205.1	188.2	-16
GGG	190.1	87.2	-14
GGA	204.2	90.1	-14
GGY	296.2	182.1	-14
GGL	246.2	132.2	-14
GGF	280.2	166.1	-14

Table S2. Repeatability test (n = 10) for six amino acids sequentially exposed to vapor additives. Sample solution: 5 μM amino acid mixture in 25% (v/v) aqueous methanol solution with 0.1% (v/v) AA. *EF*s were calculated using the following equation: $EF = I/I_0$, where I_0 is the average signal intensity without vapor, while I is the average signal intensity with vapor.

**Analyte**	**EtOH**		**IPA**		**MeCN**	
	***EF* mean**	**RSD / %**	***EF* mean**	**RSD / %**	***EF* mean**	**RSD / %**
aspartic acid	4.38	16.2	3.67	12.0	0.13	13.8
lysine	4.51	10.0	4.32	9.9	1.31	11.5
glutamic acid	5.94	13.4	3.97	9.1	0.63	11.2
histidine	3.38	10.5	3.55	10.3	1.42	10.7
tyrosine	2.56	13.0	1.54	9.0	0.19	9.2
tryptophan	2.36	11.2	1.57	10.0	0.27	9.6

**Analyte**	**PA**		**AA**	
	***EF* mean**	**RSD / %**	***EF* mean**	**RSD / %**
aspartic acid	14.96	7.3	7.79	10.6
lysine	5.60	3.6	3.03	7.6
glutamic acid	10.42	5.3	6.49	8.1
histidine	3.27	5.2	1.91	8.8
tyrosine	5.97	5.6	3.40	7.0
tryptophan	3.83	5.7	2.46	7.5

Table S3. Within-laboratory reproducibility (intermediate precision; 6 days) test for six amino acids sequentially exposed to vapor additives ($n = 6$; each day $n = 3$). Sample solution: 5 μM amino acid mixture in 25% (v/v) aqueous methanol solution with 0.1% (v/v) AA. *EF*s were calculated using the following equation: $EF = I/I_0$, where I_0 is the average signal intensity without vapor, while I is the average signal intensity with vapor.

Analyte	EtOH		IPA		MeCN	
	EF mean	RSD / %	*EF* mean	RSD / %	*EF* mean	RSD / %
aspartic acid	4.44	64.9	3.08	18.5	0.10	18.6
lysine	4.58	29.8	3.82	9.9	1.10	16.6
glutamic acid	5.99	55.1	3.32	14.9	0.49	15.2
histidine	3.47	27.1	3.09	11.8	1.16	18.3
tyrosine	2.66	50.6	1.35	14.1	0.15	11.6
tryptophan	2.50	41.0	1.43	11.2	0.21	11.5

Analyte	PA		AA	
	EF mean	RSD / %	*EF* mean	RSD / %
aspartic acid	11.76	22.4	7.38	17.8
lysine	5.26	25.9	3.27	35.5
glutamic acid	8.58	19.1	6.36	20.1
histidine	3.21	29.7	2.10	39.3
tyrosine	4.89	23.1	3.25	19.3
tryptophan	3.35	21.4	2.44	21.2

Table S4. Enhancement factors (*EF*s, $n = 3$) for six amino acids sequentially exposed to vapor additives. Sample solution: 5 μM amino acid mixture in 25% (v/v) aqueous methanol solution with 0.1% (v/v) AA. *EF*s were calculated using the following equation: $EF = I/I_0$, where I_0 is the average signal intensity without vapor, while I is the average signal intensity with vapor.

Analyte	EtOH	IPA	MeCN	PA	AA
aspartic acid	4.08±0.60	3.26±0.17	0.12±0.01	15.04±0.67	7.55±0.22
lysine	4.37±0.13	4.04±0.17	1.24±0.10	5.68±0.08	3.00±0.13
glutamic acid	5.70±0.60	3.64±0.23	0.57±0.04	10.54±0.46	6.36±0.41
histidine	3.36±0.05	3.42±0.20	1.37±0.09	3.38±0.05	1.92±0.10
tyrosine	2.45±0.27	1.44±0.07	0.17±0.01	6.17±0.22	3.35±0.16
tryptophan	2.28±0.17	1.50±0.06	0.25±0.01	3.96±0.10	2.43±0.10

Table S5. Assessment of vapor carryover (expressed as *%EF difference*; $n = 3$) by evaluating how prior vapor influences the impact of subsequent vapor on amino acid signals. The following vapor introduction sequences were tested: IPA–EtOH–IPA; MeCN–IPA–MeCN; PA–MeCN–PA and AA–PA–AA. Each vapor was sprayed for 20 s, followed by 15 s of nitrogen flushing in-between vapors to minimize residual interference. Sample solution: 5 μM amino acid mixture in 25% (v/v) aqueous methanol solution with 0.1% (v/v) AA. *%EF difference* was calculated using the formula: *%EF difference* = $(I_2\text{-}I_1)/(I_1)\times100$ where I_1 is the averaged signal of the analyte exposed to the first vapor additive in the sequence and I_2 is the averaged signal of the analyte exposed to the same vapor additive, this time, third in the sequence (after another vapor). Each analyte signal was averaged from the last 6 s of the 20-s vapor introduction.

Analyte	IPA	MeCN	PA	AA
aspartic acid	-12.79%±6.02%	15.62%±11.17%	-8.09%±2.56%	1.16%±9.56%
lysine	-16.25%±15.06%	35.39%±18.26%	-10.57%±1.72%	6.38%±17.88%
glutamic acid	-16.15%±8.15%	16.77%±10.77%	-9.14%±1.15%	1.06%±11.67%
histidine	-20.59%±10.50%	28.78%±17.29%	-15.23%±5.33%	17.43%±16.95%
tyrosine	-12.57%±16.29%	-11.22%±10.04%	-9.00%±2.43%	-3.08%±11.33%
tryptophan	-15.58%±17.55%	-11.86%±2.05%	-10.36%±4.55%	-7.15%±11.66%

Table S6. *EF*s (n = 3) for five peptides sequentially exposed to vapor additives. Sample solution: 5 μM peptide mixture in 25% (v/v) aqueous methanol solution with 0.1% (v/v) AA. *EF*s were calculated using the following equation: $EF = I/I_0$, where I_0 is the average signal intensity without vapor, while I is the average signal intensity with vapor.

Analyte	EtOH	IPA	MeCN	PA	AA
GGG	3.97±0.48	3.34±0.26	0.63±0.17	4.94±0.36	2.41±0.40
GGA	3.25±0.43	2.74±0.27	0.97±0.27	4.02±0.43	2.10±0.37
GGL	2.39±0.20	2.39±0.20	0.94±0.23	2.52±0.21	1.35±0.22
GGF	2.45±0.20	2.32±0.13	0.81±0.19	3.05±0.22	1.64±0.22
GGY	3.14±0.33	2.76±0.18	0.87±0.21	4.20±0.24	2.21±0.29

Table S7. List of *m/z* signals detected in honey sample with sequential vapor introduction using QqQ-MS in full scan mode. Only signals detected in all three sample replicates are listed. Compounds detected in both the blank and honey samples were excluded unless their signal intensity in the honey sample was at least 2-fold higher than in the blank. Compound identification was conducted using MS/MS and high resolution MS/MS analyses.

Measured *m/z* (QqQ-MS)	Measured *m/z* (Q-ToF-MS)	Theoretical *m/z*	Putative identity	Mass error (ppm)	MS/MS (NanoESI-QqQ-MS)	MS/MS (ESI-QToF-MS)
80.1						
85.1						
87.1						
99.1						
101.1						
104.2	104.1068	104.1075	choline	-7.10	Yes	Yes
113.0						
116.1	116.0704	116.0706	proline	-1.97	Yes	Yes
118.1	118.0861	118.0863	valine	-1.51	Yes	Yes
121.1						
122.2						
123.1						
127.1						
131.1						
143.2						
145.1						
147.1	147.1129	147.1128	lysine	0.49	Yes	Yes
148.1	148.0605	148.0605	glutamic acid	0.29	Yes	Yes
156.1	156.0767	156.0768	histidine	-0.49	Yes	Yes
157.1						
159.1						
162.1						
166.1	166.0863	166.0863	phenylalanine	0.13	Yes	Yes
176.1						
180.1						
181.1			glucose/ fructose		Yes	No
187.1						
198.1						
199.2						
203.1						

204.2
212.2
219.0
220.1
221.1
222.1
233.1
235.1
249.2

Table S8. List of enhanced peaks in honey samples based on full scan mass spectra (m/z 70–250). Only peaks with EF > 2 were included. Compounds detected in both the blank and honey samples were excluded unless their signal intensity in the honey sample was at least 2-fold higher than in the blank.

Vapor	Number of detected peaks	Number of enhanced peaks
Without vapor	13	-
EtOH	20	12
IPA	21	13
MeCN	11	7
PA	13	4
AA	17	9

COMPUTER CODES

Analog Discovery 2 codes for automated valve control and MS data acquisition

5-vapor sequence

```
clear();
if(!('StaticIO' in this)) throw "Please open the StaticIO instrument";
if(!('Wavegen' in this) || !('Scope' in this)) throw "Please open a Scope
and a Wavegen instrument";
const lowVoltageS = 0.2; // the solvent vapor pressure
const lowVoltageA = 0.4; // the acid vapor pressure
const highVoltage = 1.5; // nitrogen flush pressure
print("Running StaticIO script");
Wavegen.run();
Wavegen.Channel1.Mode.text = "Simple";
Wavegen.Channel1.Simple.Type.text = "DC";
Wavegen.Channel1.Simple.Offset.value = lowVoltageS;
StaticIO.run();
StaticIO.Channel1.DIO15.Mode.text = "Button";

StaticIO.Channel1.DIO15.text = "1";
print("MS");
wait(1);
StaticIO.Channel1.DIO15.text = "0";
wait(20);

StaticIO.Channel0.DIO6.text = "1";
print("6");
wait(20);
StaticIO.Channel0.DIO6.text = "0";
StaticIO.Channel0.DIO1.text = "1";
Wavegen.Channel1.Simple.Offset.value = highVoltage;
wait(15);
Wavegen.Channel1.Simple.Offset.value = lowVoltageS;
StaticIO.Channel0.DIO1.text = "0";

StaticIO.Channel0.DIO5.text = "1";
print("5");
wait(20);
StaticIO.Channel0.DIO5.text = "0";
StaticIO.Channel0.DIO1.text = "1";
Wavegen.Channel1.Simple.Offset.value = highVoltage;
wait(15);
Wavegen.Channel1.Simple.Offset.value = lowVoltageS;
StaticIO.Channel0.DIO1.text = "0";

StaticIO.Channel0.DIO4.text = "1";
print("4");
wait(20);
```

```
StaticIO.Channel0.DIO4.text = "0";
StaticIO.Channel0.DIO1.text = "1";
Wavegen.Channel1.Simple.Offset.value = highVoltage;
wait(15);
Wavegen.Channel1.Simple.Offset.value = lowVoltageA;
StaticIO.Channel0.DIO1.text = "0";

StaticIO.Channel0.DIO3.text = "1";
print("3");
wait(20);
StaticIO.Channel0.DIO3.text = "0";
StaticIO.Channel0.DIO1.text = "1";
Wavegen.Channel1.Simple.Offset.value = highVoltage;
wait(15);
Wavegen.Channel1.Simple.Offset.value = lowVoltageA;
StaticIO.Channel0.DIO1.text = "0";

StaticIO.Channel0.DIO2.text = "1";
print("2");
wait(20);
StaticIO.Channel0.DIO2.text = "0";
StaticIO.Channel0.DIO1.text = "1";
Wavegen.Channel1.Simple.Offset.value = highVoltage;
wait(60);
Wavegen.Channel1.Simple.Offset.value = lowVoltageS;
StaticIO.Channel0.DIO1.text = "0";
wait(30);
Wavegen.stop();
```

5-vapor sequence for carryover evaluation

```
clear()
if(!('StaticIO' in this)) throw "Please open the StaticIO instrument";
if(!('Wavegen' in this) || !('Scope' in this)) throw "Please open a Scope
and a Wavegen instrument";
print("Running StaticIO script");
Wavegen.run();
Wavegen.Channel1.Mode.text = "Simple";
Wavegen.Channel1.Simple.Type.text = "DC";
Wavegen.Channel1.Simple.Offset.value = 0.2; // optimize pressure
StaticIO.run();
StaticIO.Channel1.DIO15.Mode.text = "Button"
StaticIO.Channel1.DIO15.text = "1";print("MS");
wait(1);
StaticIO.Channel1.DIO15.text = "0";
wait(15);
StaticIO.Channel0.DIO5.text = "1";print("5"); // valve number
wait(20);
StaticIO.Channel0.DIO5.text = "0";
StaticIO.Channel0.DIO1.text = "1";
```

```
Wavegen.Channel1.Simple.Offset.value = 1.5;
wait(15);
Wavegen.Channel1.Simple.Offset.value = 0.2;
StaticIO.Channel0.DIO1.text = "0";
StaticIO.Channel0.DIO6.text = "1";print("6");
wait(20);
StaticIO.Channel0.DIO6.text = "0";
StaticIO.Channel0.DIO1.text = "1";
Wavegen.Channel1.Simple.Offset.value = 1.5;
wait(15);
Wavegen.Channel1.Simple.Offset.value = 0.2;
StaticIO.Channel0.DIO1.text = "0";
StaticIO.Channel0.DIO5.text = "1";print("5");
wait(20);
StaticIO.Channel0.DIO5.text = "0";
StaticIO.Channel0.DIO1.text = "1";
Wavegen.Channel1.Simple.Offset.value = 1.5;
wait(30);
Wavegen.Channel1.Simple.Offset.value = 0.2;
StaticIO.Channel0.DIO1.text = "0";
wait(30);
Wavegen.stop(); // data acqusition time 1 min
```

Vapor concentration ramp

```
clear();
if (!('StaticIO' in this)) throw "Please open the StaticIO instrument";
if (!('Wavegen' in this)) throw "Please open a Wavegen instrument";
print("Running dynamic valve control script");

// Initial setup
StaticIO.run();
Wavegen.run();
Wavegen.Channel1.Mode.text = "Simple";
Wavegen.Channel1.Simple.Type.text = "DC";
Wavegen.Channel1.Simple.Offset.value = 0.3;
StaticIO.Channel1.DIO15.Mode.text = "Button"

// Parameters
const totalTime = 2; // Total time (seconds) for the two valves to operate
const stepTime = 0.1; // Time increment for each step (seconds)
const steps = totalTime / stepTime ; // Number of steps

// Initialize timings
var vaporValveTime = 0.1; // Initial open time for DIO2 (acid)
var nitrogenValveTime = totalTime - vaporValveTime; // Initial open time
for DIO1 (nitrogen)
StaticIO.Channel1.DIO15.text = "1";print("MS");
wait(1);
StaticIO.Channel1.DIO15.text = "0";
```

```
wait(15);
for ( var i = 0; i < steps; i++) {
// Open Vapor Valve
StaticIO.Channel0.DIO2.text = "1"; //change valve number DIO
wait(vaporValveTime);
StaticIO.Channel0.DIO2.text = "0";

// Open Nitrogen Valve (DIO1)
StaticIO.Channel0.DIO1.text = "1";
wait(nitrogenValveTime);
StaticIO.Channel0.DIO1.text = "0";
var s =1
s += i
print("Step"+ s +": Vapor Valve =" + vaporValveTime + "s Nitrogen Valve ="+
nitrogenValveTime + "s");

// Update timings
vaporValveTime += stepTime; // Increase Valve for vapor time
nitrogenValveTime -= stepTime; // Decrease Valve for nitrogen time

// Ensure timings stay non-negative
if (nitrogenValveTime <0) {
nitrogenValveTime = 0;
vaporValveTime = totalTime;
StaticIO.Channel0.DIO2.text = "1"; //change valve number DIO
wait(vaporValveTime);
StaticIO.Channel0.DIO2.text = "0";
s += 1
print("Step"+ s +": Vapor Valve =" + vaporValveTime + "s Nitrogen Valve ="+
nitrogenValveTime + "s");
break; // Exit loop as Valve 2 can't open anymore
}
}
StaticIO.Channel0.DIO1.text = "1";
Wavegen.Channel1.Simple.Offset.value = 1.5;
wait(60);// final nitrogrn flushing
Wavegen.Channel1.Simple.Offset.value = 0.3;
StaticIO.Channel0.DIO1.text = "0";
wait(30);
// Stop Wavegen
Wavegen.stop();
print("Script completed");
```
